# Nuclear Mechanotransduction Across the Metastatic Cascade: Decoding Spatiotemporal Heterogeneity in Cancer Dissemination

**DOI:** 10.1002/advs.202523974

**Published:** 2026-04-02

**Authors:** Linqi Song, Jingyang Liu, Xue Wang, Minpu Zhang, Changgang Sun

**Affiliations:** ^1^ College of First Clinical Medicine Shandong University of Traditional Chinese Medicine Jinan China; ^2^ Faculty of Chinese Medicine Macau University of Science and Technology Macau China; ^3^ College of Traditional Chinese Medicine Shandong Second Medical University Weifang China; ^4^ Department of Oncology Weifang Traditional Chinese Hospital Weifang China

**Keywords:** biomechanics, nuclear mechanical response, nuclear mechanotransduction, spatiotemporal heterogeneity, tumor metastasis cascade reaction

## Abstract

Cancer metastasis is the leading cause of cancer‐related mortality, involving complex interactions between tumor cells and the mechanically heterogeneous tumor microenvironment. The cell nucleus serves as a central mechanosensor in metastasis, dynamically perceiving and responding to the spatiotemporal evolution of mechanical signals throughout the metastatic cascade. These mechanical responses, such as nuclear deformation, nuclear envelope rupture and repair, and chromatin remodeling, not only directly regulate cellular behavior but also transduce biochemical signals through mechanotransduction pathways. While studies have focused on nuclear softening, membrane rupture/repair, and mechanical memory in metastasis, a comprehensive integration of the nucleus's spatiotemporal mechanical responses across the entire metastatic process is lacking. This review proposes a “nucleus‐centered cross‐stage mechanical signal decoding” framework, highlighting how nuclear mechanosensitive components dynamically decode mechanical signals in response to changes in metastatic stages and microenvironmental features. We further explore innovative anti‐metastasis strategies targeting key nuclear mechanosensitive elements and downstream transcriptional regulators, evaluating the therapeutic potential of physical interventions at specific metastatic stages. Additionally, we discuss ongoing controversies in the field, offering a novel perspective for understanding metastasis and developing integrated therapeutic paradigms.

## Graphical abstract

Tumor metastasis results from complex interactions between cancer cells and mechanical microenvironments. We propose a “nucleus‐centered, cross‐stage mechanical signal decoding” model, highlighting how nuclear mechanosensors interpret forces at different stages. This framework informs anti‐metastatic strategies targeting nuclear mechanics and transcriptional regulators, offering insights for mechanism‐driven therapies.

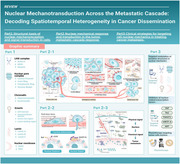

**Abbreviations**
AKT: Protein kinase B
BAD: BCL2‐associated agonist of cell death
BAF: Barrier‐to‐autointegration factor
cGAS: Cyclic GMP‐AMP synthase
CRC: Colorectal cancer
CTCs: Circulating tumor cells
CTGF: Connective tissue growth factor
CXCR4: C‐X‐C chemokine receptor 4
CYR61: Cysteine‐rich angiogenic inducer 61
DHFR: Dihydrofolate reductase
ECM: Extracellular matrix
EGFR: Epidermal growth factor receptor
EMT: Epithelial–mesenchymal transition
EMT‐TFs: EMT transcription factors
ER: Endoplasmic reticulum
ERK: Extracellular signal‐regulated kinase
FOXO: Forkhead box, class O
FSS: Fluid shear stress
GLUT3: Glucose transporter 3
HCC: Hepatocellular carcinoma
HDAC: Histone deacetylase
INM: Inner nuclear membrane
LADs: Lamina‐associated domains
lamin A/C: Lamina proteins A/C
LBR: Lamin B receptor
LINC: Linker of nucleoskeleton and cytoskeleton
LOMP: LIM Domain 7
MHC: Major histocompatibility complex
MEK: Mitogen‐activated protein kinase
MMP: Matrix metalloproteinase
MRTF‐A: Myocardin‐related transcription factor A
NER: Nuclear envelope rupture
NE: Nuclear envelope
NF‐κB: Nuclear factor kappa B
NLS: Nuclear localization signal
NPC: Nuclear pore complex
NSCLC: Non‐small cell lung cancer
ONM: Outer nuclear membrane
PD‐L1: Programmed death‐ligand 1
PDI: Protein disulfide isomerase
PI3K: Phosphoinositide 3‐kinase
ROCK: RhoA–Rho‐associated protein kinase
ROS: Reactive oxygen species
RUNX2: Regulates runt‐related transcription factor 2
SNAIL: Snail family transcriptional repressor 1
SRF: Serum response factor
STAT3: Signal transducer and activator of transcription 3
SUN: Sad1/UNC‐84
TAZ: Transcriptional coactivator with PDZ‐binding motif
TADs: Topologically associating domains
TEAD: TEA domain family member 1
TEM: Transendothelial migration
TGF: Tumor growth factor
TLR4: Toll‐like receptor 4
TPPP3: Tubulin polymerization promoting protein family member 3
TWIST1: Twist‐related protein 1
VEGF‐A: Vascular endothelial growth factor A
YAP: Yes1 associated transcriptional regulator
ZEB1: Zinc finger E‐box‐binding homeobox 1

## 1 Introduction

Tumor metastasis is the leading cause of cancer‐related mortality and is driven by a complex multistep cascade in which tumor cells detach from the primary site, invade the surrounding stroma, intravasate into the circulation, survive hemodynamic stress, extravasate into distant tissues, and ultimately colonize to form secondary foci [1, 2, 3]. This metastatic progression is driven by intrinsic genetic and epigenetic reprogramming, together with sustained bidirectional interactions with the spatiotemporal heterogeneity of the mechanical tumor microenvironment [4, 5]. Recent advances at the interface of biomechanics and cancer biology have unveiled a paradigm shift: the mechanical microenvironment functions not just as a physical barrier to dissemination, but also as a pivotal “invisible baton” that choreographs tumor cell behavior and metastatic potential [6, 7, 8, 9].

Once considered a static repository of genetic information, the nucleus is now recognized as the central hub for decoding mechanical signals [10, 11]. It not only responds passively to external forces, resulting in nuclear deformation, membrane rupture and repair, and chromatin remodeling, but also actively transduces mechanical stimuli into biochemical signals [11, 12, 13]. Mechanotransduction is primarily mediated through the nuclear–cytoplasmic shuttling of transcription factors (such as Yes1‐associated transcriptional regulator (YAP)/transcriptional coactivator with PDZ‐binding motif (TAZ), twist‐related protein 1 (TWIST1), and myocardin‐related transcription factor A (MRTF‐A), modulation of chromatin accessibility, and epigenetic modifications. These processes collectively activate key pro‐metastatic programs, including epithelial–mesenchymal transition (EMT), resistance to apoptosis, and immune evasion [14, 15, 16, 17, 18].

The precise mechanisms by which tumor cells sense, decode, and convert the heterogeneous mechanical signals encountered during the metastatic cascade into molecular events driving metastasis remain inadequately understood. Each stage of the metastatic process involves distinct spatiotemporal mechanical challenges, such as elevated extracellular matrix (ECM) stiffness in the primary tumor, fluid shear stress (FSS), and capillary constriction during circulation, as well as tissue‐specific mechanical properties in distant organs. [18, 19, 20, 21, 22]. These stage‐specific biomechanical perturbations require the tumor cell nucleus to exhibit remarkable plasticity and adaptive responsiveness. In primary tumors, nuclear softening and dynamic chromatin remodeling facilitate invasion through the dense ECM barriers [23, 24]. During hematogenous transit, cell survival depends on tolerance to extreme nuclear deformation, repeated membrane rupture–repair cycles, and the consequent activation of immune evasion and anti‐apoptotic pathways [25, 26]. Upon arrival at distant sites, the nucleus interprets local mechanical cues to sustain a pro‐metastatic phenotype, potentially through a form of “mechanical memory” that enables successful colonization and outgrowth in mechanically distinct microenvironments [27].

In recent years, several high‐quality reviews have examined nuclear mechanobiology in metastasis from distinct perspectives, including the oncogenic roles of nuclear envelope(NE) proteins, the mechanisms of nuclear envelope rupture(NER) and repair, and the potential influence of “mechanical memory” on cancer progression. However, these studies have largely remained confined to their respective subtopics—focusing either on NE components and their molecular functions or on the broader mechanical landscape of the metastatic cascade—without integrating these molecular and biophysical insights into a stage‐resolved, translationally relevant framework. A comprehensive model positioning the nucleus as a spatiotemporal decoder of heterogeneous mechanical cues across all critical steps of metastasis is still missing. To address this gap, our work makes three main contributions:

1. We propose and substantiate a nucleus‐centered, cross‐stage mechanical signal decoding framework, linking the heterogeneous mechanical environments encountered at different stages of the metastatic cascade to discrete nuclear events such as lamina remodeling, Nuclear pore complex (NPC) reorganization, and changes in chromatin accessibility. Such a nucleus‐centered, cross‐stage mechanical decoding framework fills the single‐stage focus of prior reviews and may aid in identifying the most accessible biomarker nodes.
2. We comprehensively evaluate innovative anti‐metastatic strategies targeting nuclear mechanosensitive structures and core downstream transcriptional regulators, providing a concise overview of drug development progress and translational challenges, and distinguishing between in vitro/animal and clinical evidence.
3. We critically discuss unresolved and context‐dependent phenomena—such as the bidirectional regulation of lamina proteins A/C (lamin A/C) and the distinct functional roles of the long‐presumed redundant transcription factors YAP and TAZ under different mechanical conditions—offering clear, testable hypotheses to inform future research.

In summary, this review comprehensively examines the role of the nucleus as a central mechanoregulatory hub in the metastatic cascade. Emphasizing its dual function as both a mechanical sensor and a signal transducer, we highlight the stage‐specific influence of the metastatic spatiotemporal heterogeneity of the mechanical tumor microenvironment on nuclear responses and the shaping of metastatic phenotypes. We further dissected the mechanosensitive behavior of key nuclear structures, including the NE, NPC, and linker of nucleoskeleton and cytoskeleton (LINC) complex, uncovering how distinct mechanical cues encountered at different metastatic stages differentially regulate intranuclear signaling pathways and epigenetic reprogramming, ultimately driving stage‐adaptive metastatic traits. Finally, we explored innovative therapeutic strategies that target nuclear mechanosensitive elements and downstream transcriptional regulators, along with the potential of physical therapies that modulate nuclear mechanics, to offer new perspectives and conceptual frameworks for understanding and therapeutically targeting the mechanobiology of tumor metastasis.

## 2 Structural Basis of Nuclear Mechanoreception and Signal Transduction in Cells

**FIGURE 1:**
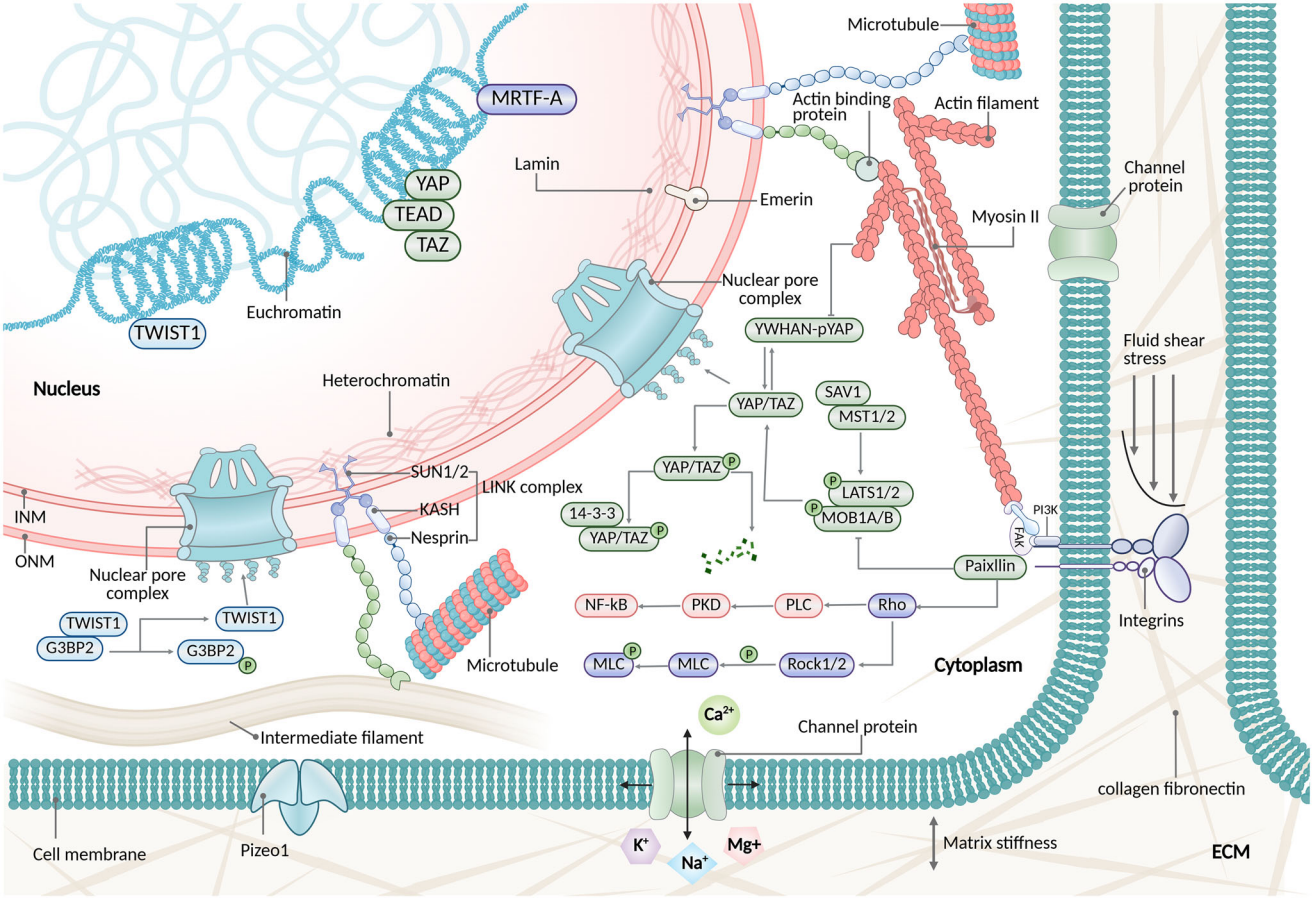
Structural basis of nuclear mechanosensing and signal transduction. The nuclear envelope (NE), comprising the outer nuclear membrane (ONM) and inner nuclear membrane (INM), serves as the first barrier for mechanical signal conversion. Its phospholipid bilayer dynamically adjusts membrane tension in response to mechanical cues. Nuclear pore complexes(NPCs), embedded within the NE, undergo conformational changes under mechanical stress, alleviating membrane tension while enhancing the nuclear import of mechanosensitive transcription factors, thereby directly coupling mechanical stimuli to gene regulation. The nuclear lamina, a dense meshwork of A‐type and B‐type (lamin B1/B2) intermediate filaments underlying the INM, provides the nucleus with its greatest mechanical strength, ensuring structural integrity. Together with INM‐associated proteins such as lamin B receptor (LBR) and emerin, it anchors chromatin to the nuclear periphery. The linker of nucleoskeleton and cytoskeleton (LINC) complex spans the NE and physically links the cytoskeleton to the nuclear interior: Nesprin proteins on the ONM (Nesprin‐1/2 bind actin filaments; Nesprin‐3, intermediate filaments; Nesprin‐1/2/4, microtubules) connect to Sad1/UNC‐84 (SUN)‐domain proteins on the INM, enabling efficient transmission of extracellular mechanical signals to the nucleus. Intranuclear chromatin organization is also critical: peripherally localized heterochromatin provides rigidity, while centrally located euchromatin maintains structural flexibility. Their dynamic interconversion modulates nuclear mechanosensitivity and facilitates adaptive transcriptional responses.

As the largest and stiffest organelle within the cell, the nucleus plays a central role in mechanosensation and signal transduction (Figure 1), owing to its unique mechanical properties and intricate molecular architecture [28]. This specialized functional capacity is derived from its distinct subcellular structure and spatial organization. The nucleus is physically connected to the plasma membrane via the cytoskeletal network, allowing it to sensitively detect and respond to mechanical cues encountered during tumor metastasis. Simultaneously, through the activation of specific signaling pathways and nuclear translocation of transcription factors, the nucleus efficiently translates mechanical stimuli into biochemical signals, thereby orchestrating key biological processes such as tumor proliferation, invasion, and dissemination [11, 29, 30, 31]. This bidirectional signaling capacity, which receives mechanical inputs and generates regulatory outputs, underscores the importance of the nucleus as a central integrator of mechanobiological information. Collectively, the NE, NPC, nuclear lamina, nuclear matrix, and chromatin constitute a sophisticated mechanotransductive network that couples mechanical sensing with gene regulatory programs [32].

### 2.1 Nuclear Membrane

The NE serves as the primary barrier for decoding mechanical signals. It is composed of a double‐layered phospholipid membrane and responds dynamically to mechanical stimuli by modulating membrane tension. Structurally, it comprises an outer nuclear membrane (ONM), which is continuous with the endoplasmic reticulum(ER), and an inner nuclear membrane (INM), which is connected to the ONM via an NPC [33]. Under resting conditions, the NE adopts a folded conformation that stores excess membrane surface area. Upon mechanical compression, these folds unfold to buffer nuclear deformation and prevent chromatin damage, facilitating the initial transduction of mechanical cues into cellular responses [34]. The NE not only serves as a mechanical barrier but also collaborates with other nuclear structures to facilitate the transmission and decoding of mechanical signals. Mechanical perturbation of the NE can lead to changes in chromatin accessibility and transcriptional activity, which will be further discussed in the following sections.

### 2.2 NPC

NPC, a critical molecular switch in mechanotransduction, is a supramolecular channel composed of approximately 30 nucleoporins, including both structural scaffolds and central transport components [35, 36]. Wolf and Mofrad demonstrated that NPC exhibits mechanically responsive conformational plasticity; under basal conditions, the central channel maintains a constricted diameter of 40–60 nm, restricting the passive diffusion of macromolecules larger than ∼40 kD [37, 38, 39, 40]. Upon mechanical stimulation, tension‐sensitive nucleoporins, such as Nup153, mediate channel dilation to 55–70 nm. This expansion not only alleviates mechanical stress on the NE but also markedly enhances the nuclear import of mechanoresponsive transcription factors such as YAP (∼65 kDa), thus directly linking mechanical cues to transcriptional regulation [41, 42].

### 2.3 LINC Complex

The LINC complex is a pivotal mediator of mechanotransduction that transmits mechanical cues from the plasma membrane to the nucleus [43]. Structurally, it comprises nesprin proteins located on the ONM, which anchor to the cytoskeleton, and Sad1/UNC‐84(SUN)‐domain proteins situated on the INM, which interact with the nuclear lamina and chromatin. This configuration forms a continuous physical linkage between the cytoskeleton and the nuclear interior, enabling the direct relay of extracellular mechanical forces to the nucleus. Notably, LINC‐mediated mechanical signal transduction occurs on a microsecond timescale—10^3^ to 10^6^ times faster than classical biochemical diffusion or signal cascade mechanisms—allowing for near‐instantaneous cellular responses to mechanical stimuli [44, 45, 46, 47]. These findings underscore the role of the LINC complex as a bidirectional integrator of mechanical cues, acting through nesprin, which channels cytoskeletal tension to modulate transcription factor import and epigenetic landscapes, whereas its structural continuity with the cytoskeleton allows for rapid and direct mechanotransmission to the interior of the nucleus [48].

### 2.4 Nuclear Fibrillar Protein

Nuclear lamina is a critical structural scaffold that preserves nuclear rigidity and mediates mechanical responsiveness [49]. It forms a dense fibrillar meshwork beneath the INM, which is primarily composed of type A and type B (lamin B1/B2) intermediate filaments. As the most mechanically robust and densely packed filamentous network within the nucleus, the nuclear lamina plays a pivotal role in maintaining the nuclear shape, mechanical stability, and structural integrity [50]. Experimental evidence demonstrates that deletion of lamin A/C markedly increases nuclear deformability, rendering nuclei more susceptible to mechanical stress and intranuclear pressure [51]. The nuclear lamina also functions as a mechanical signaling hub, which transmits cytoskeletal forces via the LINC complex and anchors chromatin through lamina‐associated domains (LADs). These interactions enable the nuclear lamina to directly regulate DNA replication and repair as well as transcriptional activity of mechanosensitive genes [52].

### 2.5 Genetic Material of Chromosome

Chromatin functions as both the ultimate effector and mechanical support element in nuclear mechanotransduction. It is a hierarchically organized and dynamic assembly of DNA and core histones (H2A, H2B, H3, and H4), with the nucleosome core particle serving as the fundamental unit [53]. Mechanical deformation of the nucleus can reorganize the chromatin architecture and modulate its interaction with the nuclear lamina, thereby activating or repressing nuclear transcription factors, indicating that chromatin exhibits an active, bidirectional mechanical response. Its multi‐tiered structural organization—from nucleosomes to 30‐nm fibers to topologically associating domains (TADs)—provides the biophysical foundation for nuclear rigidity and adaptability. Densely packed heterochromatin, typically localized at the nuclear periphery, contributes to mechanical stiffness, whereas loosely organized euchromatin, often enriched near nuclear centralis, imparts structural plasticity [54, 55, 56]. Cells dynamically convert between these chromatin states to sense and adapt to varying mechanical environments. However, chromatin also serves as a mechanosensitive substrate that receives mechanical cues transmitted via the cytoskeleton–LINC complex–nuclear lamina axis or through the mechanical redistribution of transcription factors, translating them into gene regulatory signals [57, 58]. Notably, recent studies have demonstrated that external forces can directly stretch chromatin and rapidly activate the transcription of genes such as dihydrofolate reductase (DHFR) via the F‐actin → LINC → lamin A/C → chromatin pathway, highlighting a mechanogenomic mechanism that regulates the expression of cancer‐associated genes [58].

The nuclear substructures mentioned earlier, such as the nuclear membrane, NPCs, LINC complex, nuclear lamina, and chromatin, do not operate in isolation but rather work in a coordinated manner to regulate gene expression. Mechanical stimuli first alter the tension of the nuclear membrane, thereby modulating the conformation and permeability of NPCs. This influences the nuclear‐cytoplasmic transport of transcription factors and RNA, ultimately regulating gene activation and maintenance [38, 59, 60, 61]. Specifically, mechanical stretching of the nuclear membrane can lead to the extension of tension‐sensitive proteins such as Nup153, facilitating the nuclear import of mechanosensitive transcription factors like YAP/TAZ, which connect external mechanical signals with transcriptional responses [62, 63]. Moreover, the LINC complex links the cytoskeleton with nuclear structures, transmitting mechanical stress to the nuclear lamina. This, in turn, affects the spatial organization of chromatin and epigenetic modifications, such as demethylation of H3K9me3 and increased H3K27ac, thereby activating genes associated with cell migration and invasion [64, 65, 66]. Additionally, nuclear pore proteins, beyond their structural function, also act as transcriptional co‐regulators. They directly interact with chromatin and influence epigenetic marks like H3K4me3, contributing to the remodeling of transcriptionally active regions [67, 68]. This synergy among nuclear structures enables the cell to rapidly adjust gene expression in response to mechanical stimuli, thereby regulating biological processes. In summary, mechanical input is transduced through precise adjustments of nuclear architecture, from changes in nuclear membrane tension to chromatin remodeling, ultimately achieving dynamic regulation of gene expression.

Studies have indicated the existence of a mechanical signal transduction chain within the cell. Under mechanical stretching, researchers observed the enrichment of NPCs along the “ALL line” (Actin–LINC–Lamin nuclear lines) [69]. This spatial reorganization process is dependent on the presence of SUN1. Based on these findings, the process by which the cell nucleus converts external mechanical signals into gene expression can be summarized as a continuous chain: from mechanical input to structural responses in the nucleus, leading to the final output of gene expression. Specifically, changes in matrix stiffness or intercellular tension are first perceived and integrated at focal adhesions and the cytoskeleton. These signals are then rapidly transmitted via the LINC complex to the nuclear membrane and nuclear lamina, initiating adjustments in nuclear membrane tension and nuclear deformation [57]. The coordination of nuclear structures not only regulates the entry of transcription factors through conformational changes in the NPCs but also induces chromatin remodeling and epigenetic modifications by coupling the LINC complex, nuclear lamina, and chromatin, ultimately controlling gene expression [58, 65]. This mechanotransduction pathway is fundamentally different from the mechanical receptors on the cell membrane, such as integrins, cadherins, or mechanosensitive ion channels like Piezo1. Membrane receptors primarily sense mechanical changes via the coupling of the cell membrane to the ECM, such as integrins converting ECM stiffness into biochemical cascades involving FAK and RhoA, or Piezo1 initiating Ca^2^^+^ influx through membrane stretch [70, 71, 72, 73]. These mechanosensitive molecules are primarily considered primary mechanosensors, with outputs often resulting in rapid activation of ion channels or signaling pathways, which eventually converge on the nucleus to affect gene expression. In contrast, the cell nucleus is intrinsically mechanosensitive. The NE, nuclear lamina, and chromatin are deformable mechanical structures that can directly respond to tension or compression from the cytoskeleton without relying on traditional membrane receptors or secondary signal amplification [41, 51, 74]. Notably, mechanical signals are transmitted to the nucleus via the cytoskeleton–LINC complex–nuclear membrane axis within milliseconds, inducing nuclear deformation, which is significantly faster than the biochemical cascades that rely on multistep enzymatic amplification [69, 75]. Studies have shown that mechanical signals can travel through a 50 µm fiber in approximately 2 µs, while small molecules like Ca^2^^+^ diffuse the same distance in about 25 s, and molecular motor transport takes around 50 s [76, 77].

Furthermore, unlike typical membrane mechanosensitive receptors that primarily perceive a single type of mechanical stimulus, the cell nucleus serves as a mechanical hub capable of integrating multiple mechanical cues. It responds to a variety of external forces, such as shear and compression from the ECM, and participates in the transduction and regulation of mechanical signals [11, 78, 79]. This distinguishes it from membrane receptors, which generally sense a single mechanical form. The nucleus integrates complex, heterogeneous mechanical environments, particularly in tumor biology, through mechanisms such as nuclear membrane tension changes, NPC morphological regulation, chromatin remodeling, and epigenetic modifications. These processes regulate the nuclear localization of mechanosensitive transcription factors and gene expression patterns, playing a pivotal role in cellular fate regulation, tumor cell adaptation to mechanical microenvironments, and metastatic progression [80, 81]. Additionally, the nucleus can sustain mechanical activation over time via mechanisms like persistent histone modifications, chromatin state remodeling, extracellular vesicle‐mediated signal molecule storage and delayed release, and regulation of structural proteins like nuclear lamins through long‐lived molecules like miRNAs. This long‐term memory capability is not achievable by membrane receptors, which function primarily as transient mechanosensors [27, 82, 83].

In experimental and clinical contexts, the mechanobiology of the nucleus also offers distinct advantages. Advances in technologies such as atomic force microscopy, microfluidics, image processing algorithms for cell mechanics measurement, and high‐throughput sequencing‐based chromatin structure analysis now allow precise quantification and manipulation of nuclear mechanical phenotypes [84, 85, 86, 87]. Moreover, nuclear morphology, chromatin distribution, and nuclear deformability have long been key factors in pathological diagnosis, with nuclear mechanical features like lamin A/C expression, nuclear membrane integrity, and chromatin compaction correlating significantly with the metastatic migration, circulation survival, and distant colonization abilities of tumor cells [88, 89]. Therefore, the nucleus plays an irreplaceable role in mechanosensation, signal decoding, and its clinical prognostic value.

In summary, the nucleus plays a critical role in mechanosensation and signal transduction, with its structural foundation and central position in cellular mechanics distinguishing it from integrins, Piezo, and other cell membrane mechanosensitive proteins. Unlike membrane receptors, which serve only as signal receivers, the nucleus is not only the receptor of mechanical signals but also the core decoding center for transducing mechanical information into gene expression and cellular fate regulation. The nucleus has a highly efficient mechanism for receiving and decoding mechanical signals, converting short‐term mechanical stimuli into long‐term mechanical memory. With the continued development of related experimental technologies, the clinical applications of nuclear mechanobiology hold great promise.

## 3 Nuclear Mechanical Response and Transduction in the Tumor Metastatic Cascade Response

**FIGURE 2:**
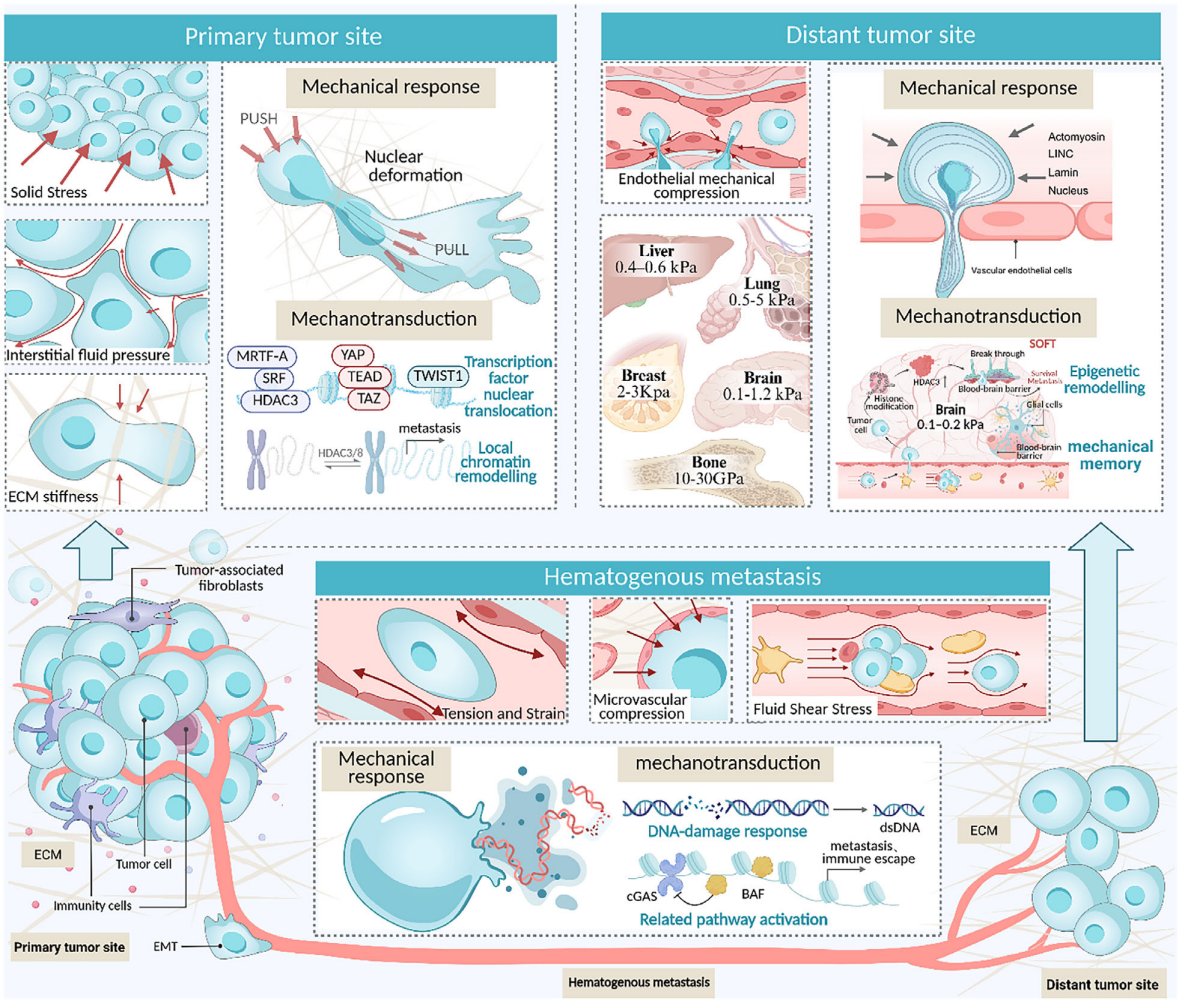
Nuclear mechanical response and transduction in the tumor metastatic cascade. The metastatic cascade in tumors can be divided into three distinct stages, based on differences in mechanical signals and the survival microenvironment: primary detachment, circulatory survival, and distant colonization. In the primary detachment stage, the primary mechanical challenges faced by the nucleus include increased extracellular matrix (ECM) stiffness, solid stress accumulation, and interstitial fluid pressure. During this stage, the nucleus dynamically adapts to mechanical stimuli through various responses such as softening and deformation. This leads to local chromatin remodeling, nuclear translocation of transcription factors, and activation of mechanotransduction pathways that drive invasive migration programs. In the circulatory survival stage, the main mechanical stresses are blood flow shear and compressive forces from lumen narrowing. Due to intense nuclear compression, the frequency of nuclear envelope rupture increases, triggering a mechanical response that activates repair pathways, such as the DNA‐damage response and cycilc GMP‐AMP synthase (cGAS)–STING. These mechanisms facilitate repair and immune evasion. In the distant colonization stage, the nucleus reshapes to adapt to the mechanical characteristics of the target organ. This transition moves from short‐term passive adaptation to long‐term alterations in nuclear chromatin stability. This process triggers persistent epigenetic reprogramming (mechanical memory), sustained yes1 associated transcriptional regulator(YAP)/transcriptional coactivator with PDZ‐binding motif(TAZ) signaling, and long‐term transcriptional programs that promote proliferation and colonization.

The metastatic cascade is a complex biological process involving multiple organs and tissues (Figure 2). Contemporary studies propose that the cascade can be stratified into three mechanobiologically distinct phases: the primary detachment stage, which includes tumor cell detachment and intravasation; the circulation survival stage, which involves cell survival during transit in the vasculature; and the distant colonization stage, which includes arrest, extravasation, and the establishment of secondary growth [1, 2, 90]. The mechanical tumor microenvironment exhibits significant spatiotemporal heterogeneity across different stages. Temporal heterogeneity manifests in the differential physical characteristics across stages. The primary tumor is dominated by solid stress and matrix stiffness, while the circulation phase is governed by fluid shear forces, and the colonization phase is marked by organ‐specific mechanical environments. Moreover, within a given stage, mechanical signals dynamically evolve as the tumor progresses [6, 91, 92] (Table 1). Spatial heterogeneity is evident in the differences between various anatomical sites and between regions within the same site. For instance, the mechanical properties of the tumor core and invasive front, as well as those of different vascular types and target organ microenvironments, are highly unevenly distributed [93, 94]. Notably, the physical microenvironment of previous stages can induce the formation of a persistent “mechanical memory” in tumor cells, which profoundly influences their subsequent biological behavior [65, 83].

**TABLE 1:** The spatiotemporal heterogeneity of the mechanical tumor microenvironment during cancer metastasis.

Tumour metastasis stage	Primary mechanical factors	Typical range of values	Mechanical source	Temporal characteristics	Refs
Primary detachment phase	ECM stiffness Solid stress Interstitial fluid pressure cellular traction force	ECM Stiffness: 4–30 kPa (or even higher) IFP: 4–100 mmHg	Collagen crosslinking activation of cancer‐associated fibroblasts tumor cell‐generated stretching and traction physical constraints caused by tumor expansile growth endothelial barrier between cells	Long‐term, sustained, and gradual	[91, 93, 95, 96, 97, 98, 99, 100, 101]
Cyclic survival phase	Blood flow shear stress	Veins: 0.5–4 dyn/cm^2^ Capillaries: 30–95 dyn/cm^2^ Arteries: 4–30 dyn/cm^2^ Lymphatic vessels: 0.64–12 dyn/cm^2^	Vascular diameter and geometry blood flow velocity, and the cyclic contraction and relaxation of the heart	Seconds‐level fluctuations High‐frequency variations Strong transience	[6, 20, 101, 102, 103, 104, 105, 106, 107, 108, 109]
Distant planting phase	Radial compression due to microvascular diameter constriction Endothelial barrier resistance Organ‐specific tissue stiffness	Brain: ∼0.1–1.2 kPa Liver: ∼0.4–0.6 kPa Bone: 10–30 GPa Lung: ∼0.5–5 kPa Breast: ∼2–3 kPa	Organ‐specific microenvironments and the shaping of the pre‐metastatic niche	Long‐term, sustained	[110, 111, 112, 113, 114, 115, 116, 117, 118]

Against this backdrop, the cell nucleus exhibits a dual response pattern: mechanical response and mechanotransduction. The mechanical response refers to passive physical alterations within the nucleus and its connections under external forces, including nuclear deformation, transient NER/remodeling, and chromatin reorganization [10, 29, 119]. These load‐dependent biophysical events form the essential foundation for subsequent mechanotransduction. Conversely, mechanical transduction involves active processes that convert external mechanical cues into gene regulatory outputs [11, 12]. This involves the nucleocytoplasmic relocalization of signaling proteins and chromatin variants through the dynamic remodeling of NPCs and force‐transmitting structures, such as LINC complexes [8, 75]. These mechanical inputs then engage mechano‐sensitive signaling pathways, including YAP/TAZ, phosphoinositide 3‐kinase (PI3K)/protein kinase B (AKT), and tumor growth factor (TGF)‐β, which orchestrate EMT, modification of cell adhesion properties, activation of transcription factors, and enhanced survival, collectively promoting progressive phenotypes at each step of the metastatic cascade.

In response to the spatiotemporal heterogeneity of the mechanical tumor microenvironment during the metastatic cascade, the mechanical response and mechanotransduction of tumor cell nuclei also exhibit significant heterogeneity. Under the selective pressures of varying microenvironmental features, tumor cells evolve diverse nuclear behaviors to meet the survival demands at each stage, progressing from “plasticity‐first” to “survival‐first” and ultimately to “adaptation and remodeling.” In the primary detachment phase, the cell nucleus softens by downregulating Lamin A/C, initiating EMT transcription to overcome the high stiffness of the ECM [24, 26, 120, 121, 122, 123]. During the circulation survival phase, nuclei increase in volume, decondense chromatin, and repair the nuclear membrane rapidly via ESCRT‐III, resisting shear stress damage, while activating cyclic GMP‐AMP synthase(cGAS)‐STING and PI3K/AKT pathways for immune evasion and anti‐apoptosis [25, 102, 124, 125, 126]. In the distant colonization phase, the nucleus adapts precisely to the target organ's matrix stiffness, integrating prior “mechanical memory” to activate organ‐specific transcriptional programs, thereby driving the formation and proliferation of micrometastases [27, 92, 127].

This article will further elaborate on the molecular mechanisms by which tumor cell nuclei mediate mechanical responses and mechanotransduction, leading to distant metastasis, focusing on the physical microenvironment characteristics at each of the three key stages of the metastatic cascade.

### 3.1 Primary Detachment Stage (Figure 3)

**FIGURE 3:**
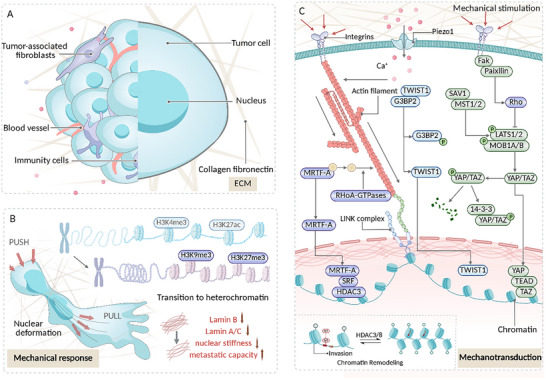
Nuclear Mechanical Responses and Signal Transduction During the Primary Detachment Stage.A. Overview of the primary detachment stage. This early metastatic phase involves three sequential steps: (1) detachment of tumor cells from the primary site, (2) invasion through the basement membrane into the surrounding extracellular matrix (ECM), and (3) intravasation into blood or lymphatic vessels. These processes are governed by mechanical cues such as ECM stiffness, solid stress, and interstitial fluid pressure. B. Nuclear mechanical responses. In response to these biomechanical stimuli, tumor cells exhibit nuclear softening—a mechanical adaptation that facilitates invasion and metastasis. This is primarily mediated by alterations in nuclear lamina composition and chromatin organization. C. Mechanotransduction mechanisms. Mechanical signals are transduced from the extracellular environment to the nucleus, where they regulate the nucleocytoplasmic shuttling of signaling molecules and induce chromatin remodeling. Key players include the twist‐related protein 1(TWIST1)–G3BP2 complex, EPHA2/LYN‐mediated phosphorylation of TWIST1, and activation of the yes1 associated transcriptional regulator(YAP)/TAZ–TEA domain family member 1(TEAD) axis. Together with epigenetic reprogramming, these pathways reinforce epithelial–mesenchymal transition (EMT), enhance cellular detachment and invasion, and drive progression to subsequent metastatic stages.

#### 3.1.1 The Spatiotemporal Heterogeneity of the Mechanical Tumor Microenvironment During the Primary Detachment Phase

The primary detachment phase marks the initial step in the metastatic cascade, where tumor cells detach from the primary site, breach the basement membrane, invade the surrounding stroma, and ultimately enter blood or lymphatic vessels. In contrast to subsequent circulatory and distant colonization phases, this stage is characterized by tumor cells primarily residing in a mechanically constrained microenvironment dominated by solid tissue structure. As the primary tumor continues to worsen, the induced mechanical tumor microenvironment becomes highly spatially heterogeneous and dynamically evolves over time, presenting a dual spatiotemporal heterogeneity that drives the subsequent metastatic cascade.

At the spatial level, the primary tumor exhibits multiscale mechanical heterogeneity. The basement membrane and ECM, composed of type I collagen and laminin, form a dense nanometer‐scale pore network (roughly 10–112 nm). In contrast, tumor cells typically exceed 10 µm in diameter, a disparity nearly 1000 times larger [128]. This considerable physical barrier forces cells to undergo extreme deformation to squeeze through and initiate invasion. Furthermore, tumor regions exhibit significant variation in ECM density, fiber orientation, and crosslinking, resulting in highly uneven local stiffness and mechanical resistance [97, 129]. Studies have shown that ECM stiffness is not only higher than in adjacent normal tissue but also exhibits a pronounced regional gradient. For example, in hepatocellular carcinoma, the ECM elastic modulus at different locations is approximately 1.1 ± 0.34 kPa, 4.47 ± 1.19 kPa, and 10.61 kPa, with a gradient increasing from the tumor center toward the invasive front. Similarly, in breast cancer, the stiffness of the tumor periphery (∼5.5 kPa) is seven times higher than in the core (∼0.74 kPa), while healthy breast tissue has a stiffness of 1.13–1.83 kPa [31, 129, 130]. Additionally, unchecked cellular proliferation leads to the gradual accumulation of solid stress within the tumor, exerting pressure on surrounding tissues and blood vessels. This alters migration paths and amplifies spatial mechanical heterogeneity by affecting local perfusion and oxygen supply [8, 130, 131].

At the temporal level, this mechanical environment also exhibits dynamic evolution rather than remaining static. In the early stages, mechanical constraints primarily stem from local barriers in the basement membrane and ECM, with their dense nanometer‐sized pores forming the primary physical structure limiting cell migration. As the tumor progresses, increased volume expansion and ECM remodeling transform the mechanical microenvironment into a complex force field driven by multiple factors. On the one hand, continued tumor cell proliferation leads to surrounding tissue compression, resulting in the accumulation of solid stress (ranging from 0.21 kPa to as high as 19.0 kPa, and even higher in human tumors) [132, 133, 134]. Notably, this stress has a dual effect: within a threshold range (∼<37 mmHg), it promotes cell migration; however, when exceeding ∼37 mmHg, it may inhibit proliferation and induce apoptosis [31]. On the other hand, increased vascular permeability and impaired lymphatic drainage significantly elevate interstitial fluid pressure (IFP), with tumor IFP ranging from 4 to 100 mmHg, well above normal tissue levels [95, 96, 99]. During intravasation, a combination of mechanical factors forms a spatiotemporal peak. Cells must traverse microvessels or lymphatic channels with diameters of only 4–9 µm, facing three simultaneous mechanical challenges: endothelial cell junctional barriers, extreme compression due to narrowing lumens (solid stress accumulation), and shear forces induced by local fluid flow [135, 136, 137].

Thus, from primary detachment to intravasation, tumor cells undergo a complex physical coupling process, initially dominated by solid barriers and progressively compounded by tissue compression and fluid shear. This continuously evolving and highly uneven mechanical background within solid tissue represents a stark contrast to the singular fluid shear environment in the circulatory phase, forming a critical transitional point from “solid” to “fluid” mechanical environments in the metastatic cascade. This spatiotemporal mechanical heterogeneity serves as the first key conversion in the metastatic cascade.

#### 3.1.2 Mechanical Response of the Nucleus During the Primary Detachment Phase

As tumor cells undergo the primary detachment phase, they encounter increasing solid mechanical challenges that accompany tumor progression. During this phase, the nucleus plays a crucial role in adaptive deformation. The nucleus has a certain degree of deformability, enabling tumor cells to survive in mechanically constrained microenvironments and successfully detach from the primary tumor. Studies have shown that tumor cells undergo significant nuclear deformation when interacting with tissue matrix and vascular basement membranes, allowing them to adapt to these physical barriers. [138, 139, 140, 141]. As early as 2004, researchers documented the deformation of endothelial cell nuclei by measuring their projected areas [142, 143]. More recently, Lele et al. simulated the fibrous obstacles encountered during cell migration, observing that the nucleus could undergo dynamic deformation, resembling a droplet with localized concavities, to facilitate cell movement [144]. Lomakin et al. have further explored the deformability of the nucleus, proposing and validating the concept of the “nuclear ruler,” stating that during confined migration, the cell is spatially confined to a size smaller than the resting nucleus, and the nucleus is deformed to unfold and stretch the nuclear membrane, triggering or modulating contractile responses that enhance propulsion to help the cell cross the narrow aperture [145]. During confined migration, cells are spatially constrained to a size smaller than the resting nucleus, and the nucleus is deformed to unfold and stretch the nuclear membrane, triggering or up‐regulating a contractile response that enhances propulsive force to help the cell pass through the narrow aperture. This observation is further evidence of nuclear deformability, and studies have shown that while passing through confined spaces, the nucleus not only undergoes morphological changes but also adaptively remodels its mechanical properties. Tumor cells with higher metastatic potential are notably softer, a phenomenon referred to as “nuclear softening.” Nuclear softening involves the coordinated regulation of multiple nuclear structures, with remodeling of the nuclear lamina protein network playing a central role [144, 146, 147, 148].

In head and neck squamous cell carcinoma, the downregulation of lamin A/C and lamin B1 expression significantly reduces the mechanical strength of the perinuclear region, thereby promoting tumor metastasis [149]. Similarly, in colorectal cancer (CRC), the phosphorylation of lamin A/C at Ser22 reduces nuclear stiffness through the direct action of Akt1, thereby enhancing cell migratory capacity in confined environments [121]. Inhibiting lamin A/C phosphorylation results in increased nuclear stiffness, which in turn hinders the migration and metastasis of CRC cells. A study by Bell et al. on breast cancer showed that reduced lamin A expression significantly enhanced nuclear deformability, enabling cancer cells to navigate physically restrictive microenvironments more effectively [146, 150]. Clinical data analysis revealed that low lamin A expression correlates with the activation of the Akt signaling pathway and poor progression‐free survival in patients [146]. Thus, the regulation of nuclear softening through lamins enhances the nuclear deformability of tumor cells in confined spaces. This reduces local stress concentration, minimizing the risk of nuclear membrane rupture, and provides the physical foundation for cancer cells to migrate through narrow ECM pores or endothelial gaps [122].

Interestingly, although lamin softening facilitates tumor cell migration within a certain range, excessive softening can compromise nuclear membrane stability, making it more susceptible to transient rupture under compression or shear forces. Recent studies have shown that under conditions of restricted migration, nuclear‐associated components such as anillin and Ect2 can redistribute to the cytoplasm and activate RhoA‐myosin II‐dependent polar contraction, thereby promoting restricted migration and invasion, a process that is further amplified by nuclear restriction‐induced rupture of the NE [13]. In addition, this rupture may lead to the detachment of chromatin fragments from the cytoplasm, which can trigger DNA damage [26]. Therefore, the dynamic regulation of chromatin state and the repair mechanism of the ruptured nuclear membrane are also crucial in the process of tumor detachment from the primary site. The tight folding of heterochromatin imparts higher mechanical strength to the nucleus, whereas a looser euchromatin configuration reduces overall nuclear stiffness. Studies have shown that short‐term mechanical stimulation (ranging from several seconds to 30 min) often results in a reduction in heterochromatin markers (e.g., H3K9me3), chromatin decondensation, and expansion of euchromatin regions. However, under the sustained mechanical load of tumor metastasis, heterochromatin markers (e.g., H3K9me3 and H3K27me3) may be repositioned or increased to enhance nuclear stiffness or adapt to mechanical stress [24]. Similarly, under three‐dimensional confined conditions, such as navigating through narrow capillaries or ECM pores, epigenetic modifications of chromatin undergo dynamic changes that influence chromatin condensation. Damodaran et al. applied a static compressive force of 1 kPa for 1 h to monolayer fibroblasts and observed an increase in the nuclear‐to‐cytoplasmic ratio of histone deacetylase 3(HDAC3) (N: C# = 124, L# = 132). This was accompanied by elevated levels of H3K9me3 and H3K27me3 (H3K9me3 N: C# = 85, L# = 118; H3K27me3 N: C# = 109, L# = 125), leading to reversible chromatin compaction [151]. This adaptive change, in coordination with the repair mechanisms of the ruptured nuclear membrane, helps to overcome the potential risk of DNA damage from nuclear softening and thus maintains the metastatic phenotype of the tumor. Although confined migration causes chromatin condensation, which reduces the accessibility of chromatin in regions near centromeres and telomeres, leading to global transcriptional suppression during this process, we observed an intriguing counter‐effect. Specifically, the accessibility of promoters associated with chromatin silencing, tumor invasion, and the DNA damage response increases. Inhibition of chromatin mechanoadaptation reduces migration speed, suggesting that inhibition of chromatin mechanoadaptation plays a crucial role in promoting confined migration [87].

It is important to note that the dynamic modulation of the mechanical properties of the nucleus does not occur in isolation, but is instead a result of the cell's precise perception and transduction of external mechanical stimuli. In this context, the role of the mechanosensitive ion channel Piezo1 is particularly crucial. Piezo1 is a large mechanosensitive calcium ion channel located on the cell membrane, capable of triggering transient Ca^2^^+^ influx in response to external mechanical stimuli such as increased matrix stiffness, fluid shear, stretch, and compression [70, 152]. Recent studies have shown that Piezo1 acts as a vital bridge between mechanical force and nuclear signaling. For instance, Deekshitha Jetta and colleagues demonstrated that Piezo1 directly alters the volume and structure of the cell nucleus by triggering Ca^2^^+^ influx under mechanical force (with a 50% reduction in planar projected area and significant nuclear volume decrease), and this process occurs independently of cytoskeletal remodeling, thereby facilitating rapid transmission of mechanical signals to the nucleus [78]. In addition to changes in area and volume, Jaquelin M. Garcia‐Castorena and colleagues found that Piezo1, upon activation by mechanical stimulation, induces F‐actin remodeling through Ca^2^^+^ influx, leading to a temporary reduction in nuclear stiffness and enabling the rapid transmission of mechanical signals to the nuclear level [153]. Furthermore, the Ca^2^^+^ influx triggered by Piezo1 also regulates the nuclear localization of YAP/TAZ and Twist1, thereby promoting EMT, enhancing ECM degradation, and facilitating tumor metastasis. As a typical mechanosensitive ion channel, the opening and signal transduction of Piezo1 can be activated by various experimental techniques, including traditional mechanical methods such as the Flexcell stretching system, PDMS mechanical stretching platforms, microfluidic systems, local compression, and point‐load systems, as well as chemical agonists like Yoda1, Yaddle1, and Yoda2 [154, 155, 156, 157, 158, 159]. In addition to conventional mechanical loading methods like strain and shear, Piezo1 can also be activated by physical field stimuli, such as ultrasound transducers, magnetically controlled nanoparticles, and magnetoacoustic coupling systems. Studies have shown that low‐intensity ultrasound (e.g., 0.5 MHz, 0.35–0.45 MPa pressure pulses) can activate Piezo1 without causing significant thermal effects, while magnetically controlled and photosensitive molecules are also enabling precise control of Piezo1 [160, 161, 162]. The development of these technologies expands the dimensions of Piezo1 activation methods, providing essential tools for further investigating this molecule and its role in the mechanical regulation of nuclear processes.

Recent evidence indicates that chromatin liquid–liquid phase separation plays an indispensable role in maintaining the metastatic phenotype during tumor cell migration through confined spaces [163, 164, 165]. Studies show that when cancer cells traverse narrow spaces, mechanical compression of the chromatin network induces the deformation, condensation, and even reorganization of molecular condensates, such as nucleoli and nuclear speckles, particularly in regions of high chromatin heterogeneity. These phase‐separated condensates significantly enhance chromatin accessibility at critical loci [166]. Notably, Xie et al. demonstrated that the pro‐metastatic transcription factor FOXM1 undergoes liquid–liquid phase separation with its target DNA, maintaining the open chromatin and super‐enhancer landscape required for metastatic growth. Thus, chromatin compaction induced by migration not only triggers traditional chromatin state adjustments but also initiates condensate‐driven chromatin remodeling and gene expression programs, providing an in vivo mechanistic link between nuclear deformation and the transcriptional programs driving invasion and metastasis [167].

#### 3.1.3 Nucleomechanical Transduction in the Primary Detachment Phase

During the primary detachment stage of tumor metastasis, tumor cells face the dual challenge of initiating EMT while overcoming the high stiffness ECM barrier and adhesive constraints. At this stage, the nucleus not only serves as the target of physical deformation but also functions as the central hub for integrating extracellular mechanical signals and initiating pro‐metastatic transcriptional programs. Tumor cell nuclei amplify the EMT signal through the activation of transcription factors such as YAP and epigenetic remodeling. These processes drive adhesion separation and invasion, laying the foundation for tumor cells to detach from the primary site and acquire high invasive potential.

The primary obstacle for tumor cells to detach from the primary site and invade the local tissue is the mechanical barrier created by solid stress from tumor volume expansion and the high stiffness of the ECM. Among these, increased ECM stiffness activates the ErbB4‐Akt1‐lamin A/C signaling pathway through the binding of integrin β1 to type I collagen and laminin, transmitting cytoplasmic tension to the nuclear membrane. This causes the phosphorylation and disassembly of the nuclear lamina protein lamin A/C at Ser22, reducing the nuclear elastic modulus and enhancing nuclear plasticity [121]. The softening of lamin A/C increases the deformability of the nucleus, enabling the cell to spread more extensively and altering the conformation of the NPC. This facilitates the translocation of YAP/TAZ from the cytoplasm to the nucleus [51]. YAP/TAZ are key effectors of the Hippo signaling pathway and core transcription factors that mediate mechanical signal transduction. Their nuclear‐cytoplasmic shuttling directly regulates the activity of downstream genes [149, 168]. Studies have shown that YAP/TAZ are predominantly localized in the nuclei of cells cultured on rigid matrices, but are mainly found in the cytoplasm of cells cultured on soft matrices. These findings have been observed in MCF10A mouse and human breast cancer epithelial cells, human mesenchymal stem cells, and IMR90 human lung fibroblasts, although the studies varied in their use of matrix stiffness, with cells cultured on soft (400–700 Pa) or rigid (>40,000 Pa) matrices [123, 169, 170, 171].

Accumulating evidence suggests that nuclear localization of YAP/TAZ plays a pivotal role in regulating tumor behavior, including cell proliferation, EMT, enhanced migration and invasion, apoptosis inhibition, and maintenance of tumor cell stemness. Notably, behaviors such as EMT, along with increased migration and invasion, are predominantly regulated by mechanical signals [149, 168]. Upon nuclear translocation, YAP/TAZ binds to the TEA domain family member 1 (TEAD) family of transcription factors, thereby activating the expression of EMT‐related genes such as connective tissue growth factor (CTGF), cysteine‐rich angiogenic inducer 61 (CYR61), snail family transcriptional repressor 1 (SNAIL), TWIST, and Zinc finger E‐box‐binding homeobox 1 (ZEB1), which enhance the migration and invasion potential of tumor cells [172, 173]. In addition, the nuclear localization of YAP/TAZ regulates the expression of cell adhesion molecules, including downregulating E‐cadherin and upregulating N‐cadherin and vimentin, further bolstering the invasive and migratory abilities of tumor cells [174, 175]. Recent work by Jiang Hongyuan and colleagues demonstrated that nuclear translocation of YAP promotes the expression of formin and myosin‐II, facilitates focal adhesion assembly, and enhances cell migration [10]. Moreover, Xiaohong et al. demonstrated that YAP/TAZ can also enter the nucleus by binding to signal transducer and activator of transcription 3 (STAT3), utilizing the nuclear localization signal of STAT3 to activate the angiogenesis transcriptional program, thereby promoting tumor metastasis [176]. Additionally, nuclear‐localized YAP initiates a positive feedback loop by activating the RhoA‐Rho‐associated protein kinase (ROCK) pathway, further enhancing cytoskeletal contractility, and increasing the deformation and invasive potential of tumor cells during metastasis [177, 178].

The TWIST1–G3BP2 axis also plays a critical role in mechanical sensing and initiation of EMT under high‐stiffness ECM conditions. As matrix stiffness increases, mechanical stimulation activates LYN kinase via the EPHA2 receptor, which then phosphorylates the Y103 site of TWIST1, releasing it from the cytoplasmic anchoring protein G3BP2. This enables TWIST1 to translocate into the nucleus, where it drives the activation of EMT‐related transcriptional programs, thereby promoting tumor invasion and metastasis [15, 16]. Kikuchi et al. established two static ECM stiffness conditions (150 Pa and 5700 Pa) using polyacrylamide hydrogels in a breast cancer model, and found that cells cultured on the stiffer matrix exhibited a markedly higher rate of TWIST1 cytoplasmic‐to‐nuclear translocation, increased Y103 phosphorylation, and greater invasive potential compared with cells cultured at 150 Pa [16]. The TWIST1–G3BP2 mechanical sensing pathway and YAP/TAZ function synergistically to provide dual protection against EMT. Notably, studies have shown that the TWIST1–G3BP2 signaling axis is specifically responsive to matrix stiffness and operates independently of cell shape, cell polarity, and adhesion junctions, in contrast to YAP/TAZ, which is sensitive to all these factors [15].

Under mechanical stimulation, MRTF family members dynamically regulate nuclear‐cytoplasmic shuttling via the RPEL domain. In a static microenvironment, cytoplasmic monomeric G‐actin binds to this domain, masking its nuclear localization signal (NLS). However, mechanical stimulation induces actin polymerization, leading to a reduction in G‐actin concentration and dissociation of MRTF‐A from G‐actin. This dissociation increases the likelihood of MRTF‐A binding to the nuclear transport receptors. In the nucleus, MRTF‐A associates with serum response factor (SRF) to activate the expression of downstream target genes, such as CTGF, collagen type I alpha 1 (COL1A1)/COL1A2, and vimentin, mediating ECM remodeling and promoting EMT, thereby facilitating tumor cell metastasis [17, 179, 180].

Once mechanical signals are transmitted to the nucleus, they not only activate transcription factors but also directly remodel the chromatin state, providing a more stable regulatory dimension for gene expression. Chromatin remodeling and epigenetic modifications are crucial during this stage. In three‐dimensional breast epithelial models, rigid matrices cause the nucleus to adopt a more “wrinkled” morphology, accompanied by an increase in inner nuclear lamina‐associated chromatin [181]. These matrices also enhance chromatin accessibility, particularly in regions rich in Sp1 binding sites, thereby activating the Sp1 transcription factor. Sp1 cooperates with HDAC3/8 to deacetylate histones, promoting the expression of EMT‐related genes and facilitating tumor metastasis [18, 181]. Kim et al. demonstrated that under conditions of high stiffness and mechanical compression, the miR‐9 promoter region in breast cancer cells undergoes increased DNA methylation. Silencing miR‐9 upregulates its target gene, vascular endothelial growth factor A (VEGF‐A), which in turn promotes angiogenesis and enhances tumor cell metastasis [182]. Additionally, short‐term exposure to high‐stiffness ECM causes chromatin to transition from a tightly packed heterochromatin state to a relatively loose euchromatin state, increasing gene accessibility. This mechanical chromatin opening offers a distinct advantage in transcriptional regulation: unlike the longer response cycles of epigenetic modifications, mechanical signals can rapidly enhance chromatin accessibility within minutes by directly stretching nucleosome fibers, allowing promoter regions of metastasis‐related genes (such as SNAI1 and SPRY2) to become accessible to transcription factors in a significantly shorter duration [24]. Furthermore, studies suggest that matrix stiffness and mechanical constraints can influence the dynamics of nuclear condensates (such as nucleosomes and nuclear speckles) by altering nuclear membrane/chromatin tension, inducing liquid–liquid phase separation [163]. This process results in the formation of membrane‐less biomolecular condensates (such as nuclear speckles and paraspeckles) within the nucleus, providing enrichment platforms for transcription factors and RNA polymerase II [183]. When these condensates are concentrated in super‐enhancer regions, they may enhance the expression of EMT‐related genes, thereby promoting EMT. Additionally, phase transitions impact the distribution of splicing factors in nuclear speckles, potentially reshaping EMT‐related splicing events and further driving the invasion and migration of tumor cells [164].

During the primary detachment stage of tumor progression, the tumor cell nucleus acts as a central hub for integrating mechanical signals. Faced with a mechanically restrictive microenvironment dominated by solid structural resistance, which intensifies as the tumor progresses, the nucleus precisely senses and transduces these mechanical inputs through multiple response mechanisms. At the mechanical response level, the nucleus adjusts its deformability, molecular composition, and chromatin state, seeking an optimal balance between “plasticity” and “stability” to maintain structural integrity while enhancing its ability to deform in high‐stiffness environments. At the mechanotransduction level, the nucleus transduces extracellular stiffness signals into specific transcriptional outputs through the nuclear–cytoplasmic shuttling of mechanosensitive transcription factors, such as YAP/TAZ, TWIST1, and MRTF‐A, along with epigenetic regulation of chromatin. This multi‐scale nuclear mechanical response network enables tumor cells to specifically recognize and adapt to the high‐stiffness microenvironment of the primary site. It ensures nuclear membrane integrity while activating pro‐metastatic transcriptional programs, laying the foundation for successful detachment from the primary tumor.

### 3.2 Cycle Survival Phase (Figure 4)

**FIGURE 4:**
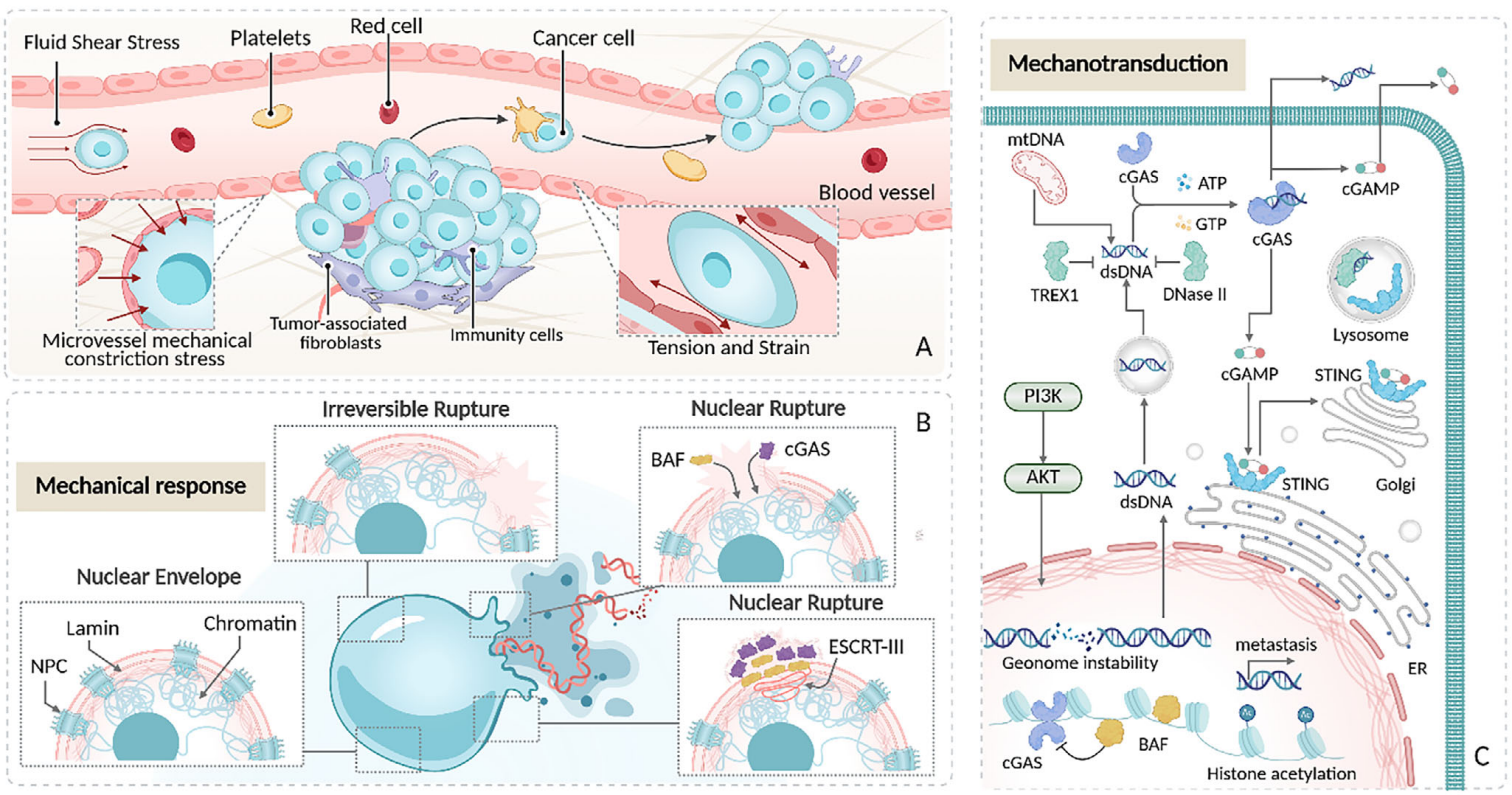
Nuclear mechanical responses and signal transduction during the circulating survival stage. (A) Overview of the circulating survival stage. This transitional phase occurs as tumor cells intravasate into the blood or lymphatic vessels and exist as circulating tumor cells (CTCs). During this brief but critical window, cells are exposed to intense biomechanical forces—chiefly fluid shear stress(FSS) from blood flow. (B) Nuclear mechanical responses. In response to circulatory mechanical stress, the nucleus undergoes pronounced deformation and softening. When these stresses exceed the structural tolerance of the nuclear envelope, rupture and subsequent repair occur. This process is orchestrated by the nuclear membrane, nuclear lamina proteins, and associated repair machinery. (C) Mechanotransduction mechanisms. Within the high‐shear environment of the vasculature, the nucleus activates distinct mechanical signaling cascades, including the cycilc GMP‐AMP synthase (cGAS)–STING and phosphoinositide 3‐kinase(PI3K)–protein kinase B(AKT) pathways. These responses promote anti‐apoptotic signaling, enhance CTC adhesion‐mediated survival, and facilitate immune evasion. Concurrently, chromatin remodeling and epigenetic modifications amplify these stress‐adaptive responses. Together, these nuclear adaptations significantly improve CTC viability and support their transition to the next metastatic stage.

#### 3.2.1 The Spatiotemporal Heterogeneity of the Mechanical Tumor Microenvironment During the Circulation Survival Phase

When tumor cells enter the bloodstream, their mechanical tumor microenvironment shifts dramatically from solid tissue constraints to a dynamic fluid shear‐dominated environment. This marks the second key spatiotemporal transition in the metastatic cascade [14, 184]. During this phase, tumor cells must survive under the continuous stress of blood flow, which is a prerequisite for successful distal colonization [102, 185, 186].

At the spatial level, there is significant fluidic heterogeneity across different vascular regions in the circulatory system. Generally, the shear stress in veins ranges from 0.5–4 dyn/cm^2^, in capillaries from 30–95 dyn/cm^2^, and in arteries from 4–30 dyn/cm^2^ [104, 105]. In hepatic sinusoids and some pulmonary vessels, where blood flow is slower, shear stress can be as low as 0.1–0.5 dyn/cm^2^ [106]. In stark contrast, shear stress can spike to several thousand dyn/cm^2^ at major vessel bifurcations or near turbulent zones around the heart [187, 188]. Lymphatic vessels exhibit generally low shear stress, averaging about 0.64 dyn/cm^2^, with peak values reaching 4–12 dyn/cm^2^ [107, 108]. The dual variation in vessel type and local flow rates creates a highly uneven shear force distribution within the circulatory environment. This spatial mechanical difference profoundly impacts the survival of circulating tumor cells(CTCs). Low shear regions facilitate cell adhesion and traversal, while high shear areas often cause cell damage [189, 190, 191, 192]. Studies indicate that only about 0.01% of CTCs survive, highlighting the circulatory system as a potent mechanical filtering barrier [193]. Though CTCs can mitigate shear stress by forming clumps or being coated with platelets, sustained fluid stress still leads to the elimination of cells with lower mechanical tolerance through mechanisms such as oxidative stress, anoikis, and immune clearance, while enriching for subpopulations with greater deformability and metastatic potential [194, 195, 196].

At the temporal level, the mechanical challenges in the circulatory phase are distinctly staged. Initially, tumor cells endure continuous FSS, which serves as the foundational mechanical background of this phase. When CTCs travel through capillaries or microvascular beds with diameters of just 4–10 µm, their size, much smaller than the lumen, forces them to temporarily lodge in place, experiencing severe radial compression induced by the narrow space [197, 198, 199]. As a result, the mechanical experience of cells shifts abruptly from sustained shear stress to instantaneous, intense compression. This rapid and extreme transition in mechanical forces gives rise to the dynamic temporal heterogeneity characteristic of the circulatory phase.

Thus, the circulatory survival phase can be viewed as a dynamic mechanical screening process, primarily driven by sustained fluid shear, with localized radial compression. This mechanical backdrop sharply contrasts with the ECM‐structure‐dominated resistance of the primary stage and provides a crucial transition for subsequent extravasation and distal colonization stages, marking an important link in the spatiotemporal heterogeneity of the metastatic microenvironment.

#### 3.2.2 Mechanical Response of the Nucleus During the Survival Phase of the Cycle

In the complex, spatiotemporally heterogeneous mechanical environment of the circulation survival stage, CTCs must endure alternating FSS and transient mechanical compression to survive. The cell nucleus, as the central hub for mechanical sensing and response, plays a pivotal role in this process. [145]. Tan et al. demonstrated that FSS increased the nuclear volume of suspended tumor cells by 2–2.5 times in an amplitude‐dependent manner, whereas the cell volume remained largely unaffected [102]. This nuclear response leads to chromatin decondensation, thereby reducing the stiffness of the tumor cells and enhancing their flexibility under shear stress. To investigate the influence of shear‐induced nuclear expansion on the survival of suspended tumor cells under FSS, osmotic stress (4% and 8% PEG) and the HAT inhibitor ANA (5 µM) were further used to inhibit this nuclear expansion process, while breast cancer cells were subjected to a shear stress of 20 dyne/cm^2^. The results showed that inhibiting the HAT‐mediated increase in nuclear size significantly reduced the survival of suspended tumor cells under shear flow. For both SKBR‐3 and MCF‐7 cells, PEG treatment decreased survival in a dose‐dependent manner by approximately 32%–60% [124].

However, chromatin decondensation induced by nuclear deformation coupled with a decline in lamin protein expression weakens the structural support of the nuclear membrane and makes the nucleus more susceptible to transient NER under extreme mechanical stress [199, 200, 201]. When the force exerted on the nuclear membrane exceeds its “safety threshold,” it bulges in localized areas and ruptures at weak points of the nuclear lamina, a process widely observed in invasive breast cancer, CRC, and mouse models [26, 125, 202]. NER exerts a bidirectional regulatory effect on the survival of CTCs. First, elevated FSS causes mechanical damage to CTCs, leading to the formation of pores in the cell membrane [203, 204]. If these pores are not promptly repaired, ion, ATP, and organelle fluxes between the intracellular and extracellular environments are dysregulated, ultimately resulting in cell death [205].

However, NER are typically localized and transient. The repair of this rupture is crucial for the survival of cells activated in circulation [26]. Upon mechanical compression‐induced rupture, Barrier‐to‐Autointegration Factor (BAF) first binds to the exposed chromatin at the rupture site, quickly recruiting ER membrane patches and initiating the initial membrane remodeling process. Subsequently, the ESCRT‐III complex, particularly the CHMP7 subunit, is activated by BAF and LIM Domain 7 (LOMP) to form polymeric helical filaments at the damage site. This process closes the NE gap via membrane contraction and budding mechanisms [125]. Meanwhile, nuclear lamin A/C undergo dynamic regulation via the phosphorylation–dephosphorylation cycle, which allows the NE to extend and cover the damaged region. Dephosphorylation induces filament reorganization, forming a reinforcing grid that maintains the mechanical stability of the repaired area. This coordinated mechanism of membrane remodeling and structural reinforcement enables the completion of NE repair within 15 min, allowing tumor cells to rapidly restore nuclear integrity during compression migration and survive in the harsh mechanical microenvironment associated with tumor metastasis [26, 125, 202].

#### 3.2.3 Nuclear Mechanotransduction in the Survival Phase of the Cycle

During the circulation survival stage of tumor metastasis, tumor cells that enter the bloodstream as CTCs must survive within a complex mechanical environment. This environment includes FSS ranging from 0.5 to 30 dyn/cm^2^, characterized by high‐frequency fluctuations and intense transient forces, as well as radial compression in microvessels. To cope with these challenges, the nucleus plays a key role. It maintains cellular activity and invasive capacity through nuclear membrane rupture and repair mechanisms. Additionally, through mechanotransduction pathways, the nucleus activates adaptive transcriptional programs, enhancing the tumor cell's ability to withstand persistent mechanical stress. These mechanisms collectively form the mechanical foundation for CTCs' survival and the maintenance of metastatic potential in the circulation.

During the survival stage, CTCs exposed to high‐intensity FSS activate the C‐X‐C chemokine receptor 4 (CXCR4) on their surface, rapidly initiating the downstream PI3K/AKT signaling pathway. This pathway promotes anti‐apoptotic and immune evasion mechanisms in tumor cells through various nuclear processes, including the regulation of key transcription factors (forkhead box, class O (FOXO), nuclear factor kappa B (NF‐κB)), apoptotic proteins ((BCL2‐associated agonist of cell death (BAD), caspase‐9), and immune checkpoints (programmed death‐ligand 1 (PD‐L1), major histocompatibility complex (MHC) I) [126, 185]. Upon AKT activation, this pathway inhibits the nuclear functions of pro‐apoptotic factors, enhances the expression of anti‐apoptotic genes, and simultaneously upregulates immunosuppressive molecules while downregulating antigen presentation. This integrated signaling pathway enhances tumor cell survival and immune escape under adverse microenvironment conditions [206]. FSS also induces histone acetylation, leading to the downregulation of pro‐apoptotic genes (Bad, Bax, p53, and p73) and the upregulation of anti‐apoptotic genes (Sod2, Bcl‐XL, and Twist1). Furthermore, shear stress relaxes chromatin, opens transcription factor‐binding sites, and amplifies anti‐apoptotic responses [102].

Additionally, under high shear stress in the bloodstream or when traversing narrow microvessels, CTCs frequently undergo transient NER, which leads to the leakage of chromatin fragments into the cytoplasm. These fragments are promptly detected by cGAS, which triggers the STING pathway and activates the secretion of type I interferons and pro‐inflammatory chemokines. This disruption of local immune surveillance enables CTCs to evade immune clearance [202]. Following cGAS‐STING activation, key transcription factors, such as NF‐κB and activator protein (AP)‐1, are activated in the nucleus, further promoting the expression of immune‐suppressive molecules such as PD‐L1 and interleukin (IL)‐10. This suppresses the cytotoxic activity of T cells and NK cells, making CTCs more resistant to immune elimination in the bloodstream [25]. Under hemodynamic shear stress, liver cancer CTCs surviving in circulation display significantly elevated expression of toll‐like receptor 4 (TLR4) and tubulin polymerization‐promoting protein family member 3 (TPPP3). In vitro, overexpression of TLR4 enhances survival under fluid shear, increases colony formation and migration, and is associated with reduced apoptosis via modulation of the p53‐Bax signaling axis. In vivo, SK‐Hep‐1 cells engineered to overexpress TLR4 or TPPP3 exhibit significantly increased metastatic colonization of both lung and liver in mouse tail–vein injection models compared with controls, consistent with the pro‐survival and pro‐metastatic roles of these genes in this model system [195].

Interestingly, we also observed the nuclear localization of YAP. However, the activating factors driving YAP nuclear translocation during the circulatory survival stage differ from those observed during the primary detachment stage [196]. Studies have shown that during the circulation survival phase, shear stress activates Piezo1, which enhances phosphorylation at the Y416 site of Src and subsequently promotes phosphorylation at the Y357 site of YAP. This cascade facilitates the nuclear translocation of YAP, which is distinct from the classical integrin‐mediated signaling pathway [207]. YAP may also upregulate anti‐apoptotic genes in the nucleus, contributing to the survival of tumor cells in the circulation and facilitating distant metastasis. Furthermore, we observed differences in the roles of YAP and TAZ in the nucleus after mechanical stimulation, challenging the traditional view that YAP/TAZ often act synergistically. This topic will be further discussed in the subsequent sections.

In vitro and animal model studies have confirmed that shear induces nuclear deformation, transient nuclear membrane rupture, chromatin remodeling, and activation of mechanosensitive pathways such as YAP/TAZ, ATR/ATM, and cGAS‐STING. A key issue, however, remains whether and how these nuclear‐level events translate into clinically measurable outcomes. Recent clinical and translational studies provide converging evidence linking these mechanisms to metastasis and survival, albeit sometimes indirectly.

First, alterations in the expression of nuclear structural proteins are associated with metastatic behavior and prognosis in human populations. For example, changes in the expression of laminin and other NE components in various tumor types correlate with higher metastatic tendencies and poorer disease‐free survival (DFS) or overall survival (OS) rates. I.M. Alhudiri et al. found that reduced or lost expression of lamin A/C was significantly associated with higher histological grade, larger tumor size, lymphovascular invasion, and the development of distant metastasis. The expression of lamin A/C was detected using immunohistochemical tissue microarray technology in 938 cases of early‐stage operable breast cancer tissues, and its relationship with clinicopathological parameters and prognosis was analyzed. Survival analysis revealed that reduced/loss of lamin A/C expression was significantly associated with shorter breast cancer‐specific survival (*p* = 0.008), which refers to the time from the date of initial surgery to death related to breast cancer [208]. In a retrospective study, clinicopathological data and FFPE tissues were collected from 370 patients with stage II–III colon cancer. Notably, among stage II microsatellite‐stable patients who did not receive adjuvant chemotherapy, the disease recurrence rate was significantly higher in the low lamin A/C expression group compared to the high expression group (100% versus 37.8%; P < 0.01) [209]. Zhengrong Wu et al. also reached similar conclusions after analyzing 126 gastric cancer clinical samples, showing that lamin A/C mRNA and protein levels were significantly reduced in gastric cancer tissues (RT‐PCR *p* = 0.011; western blot *p* = 0.036), with lower expression correlating with poorer histological differentiation (r = 0.438, ^P^ = 0.025). Immunohistochemistry showed a negative lamin A/C rate of 44.4% in tumour samples, increasing with dedifferentiation, and patients with lamin A/C‐negative tumours had significantly shorter median overall survival (26.0 ± 4.2 vs. 45.0 ± 5.5 months; *p* = 0.034). [210].

Second, the activation of typical mechanotransduction effectors in patient samples—particularly nuclear YAP/TAZ localization—correlates with increased metastatic risk and reduced survival in various cancers, consistent with their role as force‐responsive transcription mediators. Hyang Joo Ryu et al. assessed tissues from 140 invasive melanoma patients and found that melanomas with nuclear YAP showed higher mitotic activity, deeper invasion, and more frequent metastasis to distant organs compared to cytoplasmic YAP melanomas. YAP immunohistochemical staining and logistic regression analysis were performed on 140 invasive cutaneous melanoma tissue samples to assess clinicopathological factors associated with lymph node and distant organ metastasis. Results showed that melanomas with nuclear YAP expression had a significantly higher risk of distant metastasis compared to those with cytoplasmic expression (HR = 3.206, 95% CI 1.032–9.961, *p* = 0.044). Additionally, deeper tumor invasion was independently associated with both lymph node metastasis (HR = 1.279, 95% CI 1.076–1.520, *p* = 0.005) and distant organ metastasis (HR = 1.197, 95% CI 1.016–1.409, *p* = 0.031) [211]. In a retrospective analysis of 455 breast cancer specimens from Severance Hospital, Yoon Jin Cha et al. demonstrated that high nuclear YAP1 expression was not only associated with hormone receptor negativity and aggressive tumor behavior (including lymph node metastasis and high Ki‐67 labeling index), but also correlated with inferior distant metastasis‐free survival and disease‐free survival. [212].

Third, alterations in the expression of markers associated with nuclear membrane rupture and repair are closely related to metastasis and survival in cancer patients. Several IHC, TCGA, and cohort analyses have shown that high expression of BANF1/BAF is commonly associated with poor prognosis in various cancers, including gastric and triple‐negative breast cancer [213, 214]. Junjun Li et al., in a study of 132 post‐surgical gastric cancer samples, found that BANF1 mRNA expression was significantly higher in tumor tissues compared to normal tissues. Additionally, BANF1 expression correlated with patient age, tumor differentiation, and invasion depth, with high BANF1 expression linked to shorter survival times compared to patients with low expression [213]. Furthermore, as key effectors of nuclear membrane rupture and repair, several clinical studies have focused on ESCRT family proteins, such as NLRP3. Jisup Kim et al. analyzed 351 endometrial cancer patient samples and found that high expression of NLRP3 was associated with poor recurrence‐free survival (RFS), particularly in advanced stages of endometrial cancer [215].

In conclusion, these clinical and translational data, specifically clinical cohort studies linking altered nuclear structural proteins (e.g., lamin A/C), mechanotransduction effectors (e.g., nuclear YAP/TAZ localization), and nuclear membrane rupture/repair markers (e.g., BANF1 and NLRP3) to metastasis, disease recurrence, and overall patient survival—support the idea that shear‐ and compression‐induced changes in nuclear mechanisms can significantly influence metastatic progression and survival outcomes. However, we emphasise that many clinical studies report evidence of correlation rather than causation and that direct measurement of nuclear membrane rupture and repair in patients remains technically challenging. Therefore, we suggest that future clinical cohorts integrate newer assays for nuclear integrity, chromatin accessibility, and mechanotransduction characteristics to allow for more precise prospective validation of mechanically mediated prognostic markers.

In the circulation survival phase, the tumor cell nucleus acts as the central hub for integrating mechanical signals. Faced with the fluid shear‐dominated environment and radial compression from microvessels, the nucleus precisely perceives and transduces these mechanical inputs through complex response mechanisms. Mechanically, the nucleus adjusts its size, chromatin state, and membrane integrity, striking a dynamic balance between “deformability” and “structural stability.” In terms of mechanotransduction, the nucleus utilizes mechanosensitive signaling pathways such as cGAS‐STING, PI3K/AKT, and YAP, alongside chromatin epigenetic modifications, to convert extracellular mechanical stimuli into specific transcriptional outputs, regulating genes involved in anti‐apoptosis, adhesion, survival, and immune evasion. This nuclear mechanobiological network enables tumor cells to adapt to the dynamic mechanical environment of the circulatory system, maintaining nuclear plasticity and functional integrity while activating pro‐survival transcription programs. These processes lay the foundation for subsequent extravasation and distant colonization.

### 3.3 Circulation Survival Stage (Figure 5)

**FIGURE 5:**
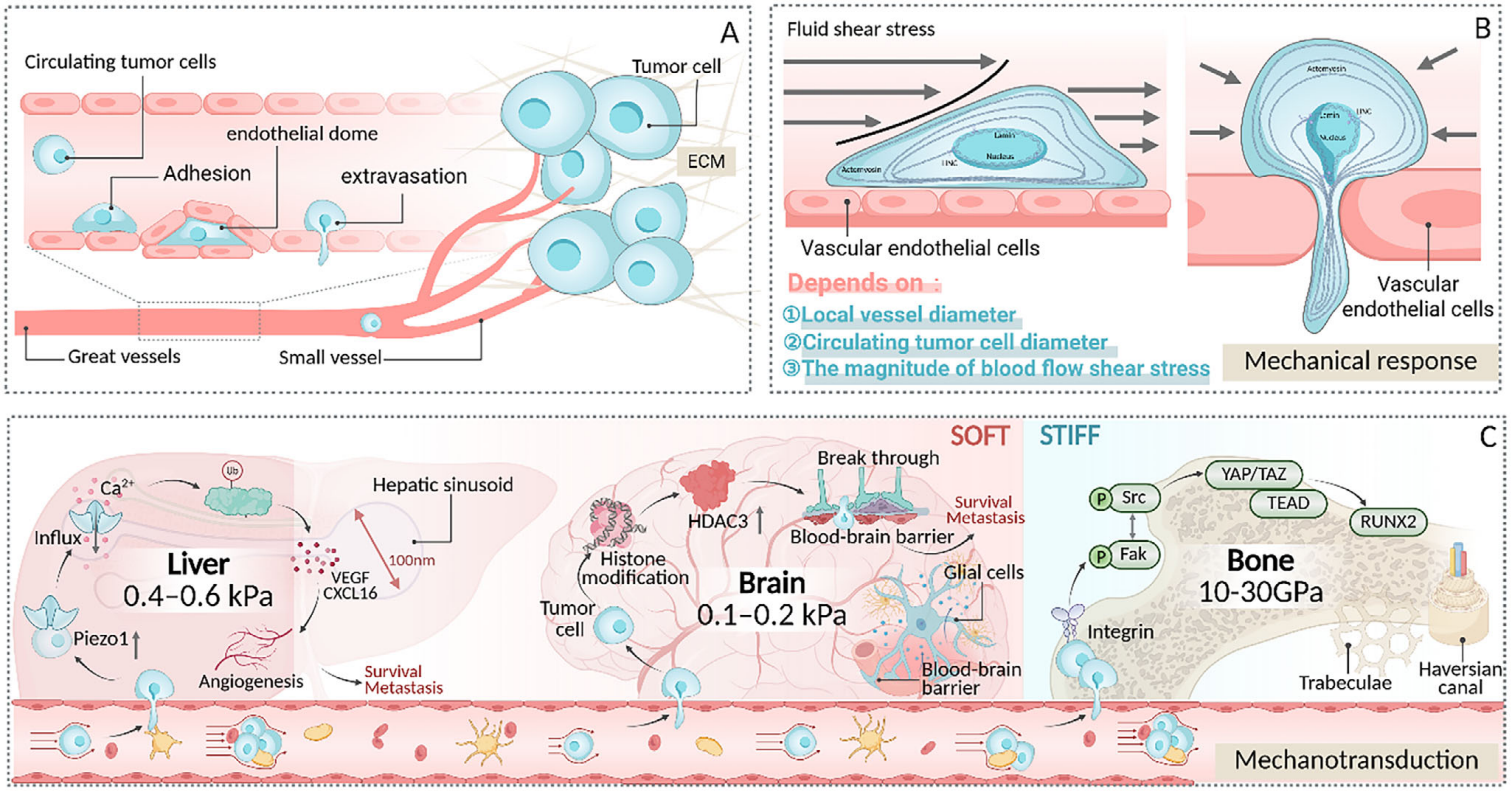
Nuclear Mechanical Responses and Signal Transduction During the Distant Colonization Stage. A. The distant colonisation phase comprises two critical steps: tumor cells traverse vascular or lymphatic barriers (extravasation) and subsequently survive and proliferate within distant tissues to form metastatic foci. This process is regulated by mechanical signals such as blood flow shear stress and stromal stiffness. B. Nuclear mechanical responses. While the types of mechanical stimuli resemble those encountered during primary detachment, the magnitude and context vary significantly across different target organs. The nucleus adapts through organ‐specific activation of mechanosensitive structures, enabling successful colonization. C. Nuclear mechanotransduction. During this phase, nuclear mechanotransduction integrates organ‐specific mechanical structures with “mechanical memory,” adapting to the local niche through mechanisms such as yes1 associated transcriptional regulator(YAP) nuclear localization and epigenetic modifications. These processes enable tumor cells to remodel their microenvironment, establishing a positive feedback loop that supports sustained proliferation and metastatic growth.

#### 3.3.1 The Spatiotemporal Heterogeneity of the Mechanical Tumor Microenvironment During the Remote Planting Phase

When CTCs arrest in distal microvascular beds and initiate extravasation, their mechanical tumor microenvironment undergoes a fundamental transformation: shifting from the dynamic fluid shear forces of the circulatory phase to a complex solid resistance field composed of both vascular structural barriers and organ‐specific ECM properties [216, 217, 218]. Unlike the fluid shear environment of circulation, this new environment reintroduces solid constraints and superimposes organ‐specific matrix mechanical characteristics, marking the third critical transition in the metastatic cascade.

Spatially, the process of tumor cell extravasation and colonization is regulated by a hierarchy of mechanical factors. Initially, extravasation is limited by structural differences within the local vasculature. CTCs are often trapped at capillary bifurcations or in smaller microvascular regions, where blood flow slows, and radial compression is sustained [219, 220]. Additionally, the strength of endothelial junctions and the thickness of the basement membrane vary significantly across organs: liver and bone marrow vessels are typically porous or sinusoidal, with loose endothelial connections that facilitate tumor cell penetration, while microvessels in the brain and lungs are narrower and more rigid, imposing greater demands on cell deformation and transendothelial migration(TEM). These variations create spatially heterogeneous structural resistance that tumor cells must overcome to cross the endothelium [94, 193, 221, 222]. Once extravasation is completed, cells must adapt to the organ‐specific mechanical environment. Different target organs exhibit considerable differences in matrix stiffness, fiber structure, and tissue elasticity. For instance, brain tissue is very soft (elastic modulus ≈ 0.1–1.2 kPa), while liver, breast, and lung tissues are somewhat stiffer (≈ 0.4–0.6 kPa, ≈ 2–3 kPa, ≈ 0.5–5 kPa), and bone has stiffness values up to 10–30 GPa [110, 111, 112, 113, 114, 115, 116, 117]. These orders‐of‐magnitude differences in stiffness create vastly different “soil” for colonization, profoundly influencing tumor cell mechanical adaptability and subsequent proliferation [223]. Furthermore, tumor cells do not merely passively adapt but actively remodel the local mechanical environment. For example, exosomes and growth factors secreted by the primary tumor activate fibroblasts and macrophages in distant tissues, promoting collagen deposition and matrix remodeling, which increases local stiffness and enhances vascular permeability, thereby creating a “pre‐metastatic niche.” This organ‐level mechanical remodeling, combined with the inherent differences in matrix fiber alignment and pore structure, leads to a multi‐scale spatial heterogeneity in distant tissues, providing a highly uneven mechanical backdrop for tumor cell colonization [118, 224].

Temporally, the mechanical stimuli during this stage exhibit clear sequential changes. Initially, during extravasation, cells mainly experience sustained vascular compression and resistance from the endothelial structure. Upon entering the tissue stroma, they face local matrix constraints and spatial limitations. As micro‐metastases begin to form and proliferate, local solid stress and matrix remodeling accumulate, further altering the tissue's mechanical state [131, 193, 219]. This continuous transition—from acute vascular obstruction and trans‐wall migration to chronic tissue remodeling—introduces significant temporal dynamics into the colonization phase.

In conclusion, the extravasation and distant colonization stages represent a continuous process transitioning from vascular structural resistance to organ‐specific solid constraints. This mechanical backdrop is distinct from the solid tissue constraints dominant in the primary stage and the dynamic fluid shear forces central to the circulatory phase. As the final step in the metastatic cascade, this phase presents a complex mechanical landscape shaped by the interplay of multiple mechanical characteristics and organ‐specific factors. It is not only the ultimate destination for circulating survivors but also the final test of their mechanical adaptability.

#### 3.3.2 Mechanical Response of the Nucleus at the Stage of Distant Colonization

Interestingly, during the colonization stage, tumor cells exhibit mechanical responses similar to those observed during primary detachment, such as changes in nuclear stiffness and morphology as well as nuclear membrane rupture and repair. This similarity likely stems from comparable mechanical signals encountered during the two stages. However, owing to differences in the magnitude of these mechanical forces, tumor behavior, and the specific environment at the colonization site, the nuclear mechanical response at this stage differs in some respects from that observed during primary detachment.

First, CTCs must adhere to the vascular wall to extravasate. At this stage, two primary mechanisms facilitate tumor cell retention: physical occlusion and cell adhesion [225, 226]. The likelihood of these mechanisms depends on (1) local vascular diameter, (2) cell diameter, and (3) blood flow shear stress [90, 227]. Owing to the larger size of tumor cells compared to blood vessels and the fact that CTCs often exist as cell clusters during metastasis, they are more prone to retention and experience significant compression within microvessels. Owing to its physical properties, the cell nucleus serves as the primary mechanical bottleneck for migration [228]. Studies have shown that reduced nuclear stiffness and deformation significantly lower the likelihood of cell obstruction, thereby facilitating the passage of CTCs through narrow vessels. Computational models further demonstrate that “softer” CTCs are more likely to come into contact with the vascular wall and adhere firmly within microvessels, whereas “harder” CTCs are pushed away by red blood cells and struggle to adhere. Based on the shear modulus, three levels of CTC stiffness are defined: CTCs softer than red blood cells (k_ctc_ = 0.5k_rbc_, labeled as ‘SOFT’), CTCs stiffer than red blood cells (k_ctc_ = 10k_rbc_, labeled as ‘RIGID’), and CTCs with deformability equal to that of red blood cells (k_ctc_ = k_rbc_, labeled as ‘EQUAL’) [229]. Recent advancements in dual‐fluorescent cell lines enable more detailed imaging of CTC deformation within capillaries. Using this technology, Yamamoto observed that fibrosarcoma cells traversing the lung and brain microvessels underwent extreme elongation of both the nucleus and cytoplasm [230]. This deformation depends on the coordinated interactions between several nuclear structures, including the nuclear membrane, LINC complex, nuclear lamina, and nuclear skeleton [188]. During the distant colonization phase, these complexes couple the nucleus to the cytoskeleton via actin filaments attached to the nuclear membrane. During migration through a narrow 3D microenvironment, the nucleus experiences force from myosin‐actin contraction, promoting nuclear deformation and facilitating tumor cell displacement [231].

In addition, the cell nucleus exhibits adaptive changes in response to the mechanical properties of different colonization sites. For example, in the relatively soft brain, tumor cells tend to downregulate the expression of nuclear lamina proteins (such as lamins A/C), thereby softening the nucleus and facilitating migration and colonization within soft matrices [51]. In contrast, when colonizing harder tissues, such as bone, tumor cells may upregulate the expression of nuclear lamina proteins to increase nuclear stiffness, thereby adapting to the mechanical stress posed by rigid matrices.

#### 3.3.3 Nuclear Mechanotransduction in the Remote Colonization Phase of the Cell

Upon reaching distant tissues and initiating extravasation to form micrometastases, the mechanical microenvironment of CTCs undergoes a dramatic shift. The fluid shear‐dominated environment of the circulation transitions into the complex mechanical landscape where vascular structural resistance gives way to organ‐specific solid tissue constraints during colonization [6]. Significant differences in matrix stiffness, microtopology, and hemodynamics across target organs induce strong organ‐specific mechanical signaling within the nucleus, often amplified by local stress factors such as hypoxia and nutrient deprivation [232]. In this phase, tumor cells must not only adapt to mechanical changes through cellular responses but also integrate the “mechanical memory” from the circulation stage with the mechanical signals from the target organ microenvironment. This is achieved through chromatin remodeling and transcriptional reprogramming, initiating adaptive programs necessary for successful colonization [92].

Once tumor cells are captured and adhere to the vascular wall under mechanical signal stimulation, they either undergo TEM to cross the basement membrane or form endothelial domes around CTCs, regulated by FSS, to colonize secondary sites [189, 227]. Although endothelial dome formation represents a passive adaptive response, TEM is a critical active process for tumor cells to achieve extravasation and distant metastasis, and plays a dominant role in the spatial breakthrough of metastasis. Jacky G. Goetz and colleagues leveraged the multiple advantages of zebrafish embryos to identify a critical threshold in hemodynamic characteristics (400–600 mm/s). This threshold is closely associated with the stable arrest of CTCs in permissive vascular regions, serving as a prerequisite for tumor cell TEM and subsequent metastasis. The study further indicates that blood flow shear stress significantly influences tumor cell TEM by modulating mechanical signal transduction within the cell nucleus [189]. Using a zebrafish model, Ma et al. demonstrated that reactive oxygen species (ROS) levels in tumor cells were upregulated under FSS (10–15 dyn/cm^2^), activating the mitogen‐activated protein kinase (MEK)/extracellular signal‐regulated kinase (ERK) signaling pathway and potentially inducing ERK nuclear translocation to regulate the expression of genes such as matrix metalloproteinase (MMP) and intercellular adhesion molecule 1 (ICAM‐1), thereby promoting TEM [216]. Another study found that when the shear rate exceeds 400 s^−^^1^, typical of physiological shear conditions, the nucleus coordinates with cytoskeletal morphological changes via the linker complex, inducing tumor cells to form pseudopodia, which further facilitates TEM [233].

Moreover, mechanical induction during tumor metastasis can be retained through mechanical memory and biophysical adaptation, allowing tumor cells to maintain their migratory, anti‐apoptotic, and stemness properties via mechanisms such as chromatin remodeling and transcriptional network reprogramming. This effect extends to the colonization of distant organs [27]. The term “mechanical memory” was first introduced in 2012 by Balestrini et al., who discovered it in fibroblasts [234]. This concept posits that cells alter their phenotype in response to specific physical microenvironments and that this phenotype can persist even after the original physical stimulus is removed, with the cells subsequently exposed to a new environment [27]. In the final stages of the tumor metastasis cascade, the activation of resident mechanical memory becomes a crucial factor in tumor colonization. Successful distant colonization requires the fulfillment of three key prerequisites: (i) reinitiating tumor growth through cancer stem cell self‐renewal, (ii) adapting and establishing organ‐specific colonization strategies, and (iii) creating a supportive microenvironment for metastatic colonization. Notably, the adaptation and establishment of organ‐specific colonization strategies are closely linked to the activation of mechanical memory [235].

The mechanical memory activated by differences in stiffness and topology across various target organs can adapt and establish organ‐specific colonization programs. Significant differences in the tissue mechanical properties of metastatic target organs are evident. (Table 2) For instance, normal brain tissue is extremely soft, containing abundant glial cells and a network of ECM, with a tight blood–brain barrier and very low collagen content, yielding a Young's modulus of approximately 0.1–1.2 kPa [110]. Liver tissue, also relatively soft, features a unique hepatic sinusoidal system, with fenestrated endothelial cells having pores around 100 nm in size, yielding a Young's modulus of 0.4–0.6 kPa [111, 112]. Pulmonary tissue is composed of a porous network of air sacs rich in elastic fibers and thin membranes. The large air spaces in alveoli render the lung tissue more “compliant,” with an elasticity modulus of approximately 0.5–5 kPa [115, 116]. In the softer brain tissue environment, cancer cell nuclei typically adopt a near‐spherical shape with low nuclear lamina tension. In this context, mechanotranscription factors such as YAP/TAZ are less likely to localize to the nucleus, and stress signals are primarily mediated through pathways like histone deacetylation. Tang et al. showed that the soft microenvironment of tumors in situ can inhibit cytoskeletal–nuclear mechanotransduction, activating HDAC3 and instilling mechanical memory. This allows tumor cells to retain brain metastatic characteristics even after encountering different mechanical microenvironments during metastasis [236]. In liver tissue, although low stiffness is generally unfavorable for Piezo1 expression, mechanical memory allows tumor cells pre‐cultured on high‐stiffness matrices to retain Piezo1 nuclear localization in soft environments. This maintains expression of pro‐angiogenic factors (VEGF and CXCL16), which enhances the permeability of hepatic sinusoidal endothelial cells, supporting the survival of micro‐metastatic foci [237, 238]. Conversely, bone tissue, composed of mineralized collagen with Haversian canals and bone marrow cavities, exhibits extremely high stiffness, reaching values of 10–30 GPa [117]. In the highly rigid bone environment, cell nuclei become compressed, nuclear lamina tension increases, and LMNA (lamin A/C) expression is upregulated, facilitating nuclear translocation of YAP/TAZ and activation of downstream genes. For example, in bone marrow, matrix stiffness enhances YAP/TAZ nuclear translocation via the integrin β1‐FAK‐Src pathway, leading to sustained YAP accumulation in the nucleus. These nuclear stress and chromatin state alterations are likewise “remembered.” The persistent accumulation of YAP regulates runt‐related transcription factor 2(RUNX2) and downstream osteogenic genes, promoting tumor cell resistance to immune surveillance in the bone marrow microenvironment and accelerating proliferation [239, 240, 241]. Watson et al. also established a “mechanical modulation” score in breast cancer cells and found that the mechanical memory features induced by high stiffness pre‐treatment correlate with bone metastasis in patients [239].

**TABLE 2:** Quantitative induction of mechanical signals, nuclear mechanical conduction, and experimental models in cancer extravasation and colonization.

Tumour metastasis Stage	Primary mechanical factors	Quantitative parameters		Measurement method	Primary effect	Experimental model	Refs
extravasation	Blood flow shear stress	Shear stress during cycling: 0.5–30 dyn/cm^2^		Ultrasound Doppler/vector flow imaging(in vivo) Phasecontrast MRI + Wall Shear Stress Quantification(In vivo)	1. Transient changes in nuclear stiffness and morphology 2. ctivation of nuclear membrane rupture and repair mechanisms 3. Nuclear localization of transcription factors 4. Adapt to mechanical signals within blood vessels to survive.	Microfluidic vascular models(Ex vivo) Zebrafish models(In vivo) Chicken chorioallantoic membrane models(In vivo)	[103, 189, 199, 242, 243, 244, 245, 246, 247, 248, 249, 250, 251, 252, 253, 254]
	Localised compression of the microvasculature	Microvascular diameter: 4–10 µm (smaller than CTC diameter ∼7–30 µm)		Multiphoton microscopy imaging, confocal microscopy imaging + software measurement(Ex vivo)			
colonization	Target organ tissue stiffness	Brain	∼0.1–1.2 kPa	Magnetic resonance elastography(In vivo) AFM micro‐indentation(Ex vivo)	1. Persistent alterations in nuclear rigidity and morphology 2. Nuclear localisation of transcription factors 3. Mechanisms of epigenetic remodelling 4. Initiation of mechanical memory enabling organ implantation	3D Simulated Brain Stiffness Matrix Gel(0.5 mg/ml Collagen I + 3 mg/ml Matrigel+ 3.3 mg/ml Hyaluronic Acid)	[11, 27, 83, 110, 111, 112, 117, 236, 238, 239, 240, 241, 255, 256, 257, 258, 259, 260, 261, 262, 263, 264, 265, 266, 267, 268]
		liver	0.4–0.6 kPa	Magnetic resonance elastography(In vivo) Ultrasound shear wave elastography (In vivo) AFM micro‐indentation(Ex vivo)		biomimetic liver tumor‐on‐a‐chip model(decellularized liver matrix (DLM)+microfluidic chip)	
		bone	10–30 GPa	Uniaxial compression / tension(Ex vivo) Three‐point bending(Ex vivo) Nanoindentation(Ex vivo)		(Adjustable aperture/stiffness+PDA surface modification)	

**TABLE 3:** Clinical strategies for targeting the cell nucleus in treating cancer metastasis.

Belongs to	Drug name	Target	Cancer type	Mechanism of action	Research phase	RefsClinicalTrials.gov ID
	Betulinic acid	lamin B1	Pancreatic cancer	Targets lamin B1 and disrupts the nuclear lamina structure, weakening the proliferation, invasion, and tumorigenicity of pancreatic cancer cells.	In vitro/in vivo	[283]
	Mevinolin	lamin A/C	Ewing's sarcoma	Promotes the accumulation of prelamin A and upregulates the expression of mature lamin A by inhibiting HMG‐CoA reductase and interfering with the farnesylation process of the precursor protein prelamin A. This significantly reduces the migration and invasion abilities of tumor cells.	In vitro	[284]
Targeted modulation of sensitive structures in nuclear mechanics	5‐Azacytidine	lamin A/C	Ewing's sarcoma	Increases the expression of the LMNA gene and the levels of lamin A/C, and reduces cell migration.	In vitro	[284]
	Lonafarnib/ Lonaf arnib+KRAS‐G12C	lamin A/C	Cervical cancer	Leads to the accumulation of prelamin A by inhibiting its farnesylation and upregulates the expression of mature lamin A. Studies have shown that Lonsurf can synergize with KRAS‐G12C inhibitors to enhance the antitumor effect.	In vitro/in vivo/Phase II clinical	[285, 286] NCT00773474
	A salt molecule of a bis Schiff base, M2	Link complex	Multiple cancers	Stabilizes the G‐quadruplex structure in the promoter region of LINC00273 and inhibits its expression, thereby significantly reducing the migration and invasion abilities of tumor cells.	In vitro/in vivo	[287]
	MK2206	Lamin A/C	Lung cancer	Inhibits the phosphorylation of lamin A at Ser390 by AKT2, thereby suppressing nuclear deformation and genomic instability, restoring the integrity of the nuclear skeleton, and inhibiting the migration and invasion abilities of tumor cells.	In vitro/in vivo/Phase I	[288] NCT01071018
	Verteporfin	YAP	Breast cancer	Inhibits YAP‐mediated tumor proliferation and metastasis by eliminating the interaction between YAP and TEAD	In vitro/in vivo/Phase I/IIa	[289] NCT02872064
	Okadaic acid	YAP/Taz	—	Inhibits protein phosphatases PP1 and PP2A, increasing the phosphorylation of YAP/TAZ and transferring YAP/TAZ to the cytoplasm, thereby inhibiting tumor metastasis.	In vitro	[290, 291, 292]
Targeted regulation of nuclear‐related transcription factors	CA3	YAP/Taz	Mesothelioma	Inhibits YAP‐mediated TEAD transcriptional activity, reducing the expression of downstream target genes and decreasing the migration and invasion of tumor cells.	In vitro	[293]
	JM7	YAP/Taz‐TEAD	Mesothelioma, breast cancer, and ovarian cancer	Specifically inhibits the spontaneous palmitoylation of TEAD1‐4, leading to unstable degradation of TEAD, thereby suppressing the transcriptional activity of YAP and inhibiting tumor proliferation, colony formation, and migration.	In vitro	[294]
	TY‐0584	YAP/Taz‐TEAD	Mesothelioma, head and neck cancer	Exerts antitumor effect by inhibiting the palmitoylation of TEAD and blocks the transcriptional activity of YAP/TEAD.	In vitro/in vivo	[295]
Targeted regulation of nuclear‐related transcription factors	SHPTK2 PND1186	YAP	Cholangiocarcinoma	Inhibits p‐YAPY357 to suppress YAP activation and nuclear localization, thereby inhibiting tumor occurrence and progression.	In vitro/in vivo	[296]
	Harmine	TWIST1	Lung cancer	Promotes the specific degradation of the TWIST1‐E2A heterodimer, inhibits the stability and nuclear activity of TWIST1, and reduces tumor metastasis.	In vitro/in vivo	[297]
	CCG‐1423	MRTF‐A	Melanoma	Combines the nuclear localization signal region of MRTF‐A and blocks its interaction with importin α/β1, thereby inhibiting the nuclear transport of MRTF‐A.	In vitro	[298, 299]
	CCG‐203971	MRTF‐A	Melanoma		In vitro	[299]
	CCG‐222740	MRTF‐A	Melanoma		In vitro	[300]
	Camonsertib (RP‐3500)	ART	Multiple cancers	May induce tumor cell death and thereby inhibit tumor metastasis by enhancing DNA damage.	In vitro/in vivo/Phase I/IIa	[301] NCT04497116
	Berzosertib	ART	Multiple cancers	Leads to the accumulation of DNA damage by interfering with DNA damage detection, reducing the vitality of tumor cells, and thus may inhibit metastasis.	In vitro/in vivo/Phase I/II	[302] NCT04266912
	Tumor electric field stimulation	Microtubules, nuclear membrane, cytoskeleton	Multiple cancers	Interferes with mitosis, induces nuclear membrane rupture, activates related signaling pathways, and inhibits cell migration and invasion.	In vitro/in vivo/Phase II	[303] NCT04902586
	High‐intensity focused ultrasound	Nucleus membrane, DNA repair pathway	Multiple cancers	Nuclear membrane rupture is induced by thermal effect and mechanical force, which leads to DNA damage and inhibition of metastasis.	In vitro/in vivo/Phase II	[304] NCT00008437
Physiotherapy	Focused ultrasound and sensitizing chemicals	Apoptosis‐related pathways in the nucleus	Prostate cancer	The ultrasound and the chemically induced stress significantly upregulate the intranuclear stress signals of cancer cells, inducing apoptosis and reducing the metastatic phenotype.	In vitro/in vivo/Phase I	[305] NCT04796220
	Low‐intensity pulsed ultrasound	Cytoskeleton, cGAS‐STING. Nuclear membrane	Prostate cancer	It destroys the cytoskeleton of cancer cells, leading to the loss of nuclear membrane integrity and activating the cGAS‐STING immune pathway, thereby inhibiting tumor metastasis.	In vitro	[306]
	Gold nanoparticles targeting the nuclear membrane	Lamin A/C	Ovarian cancer	Gold nanoparticles with nuclear localization signals can accumulate near the nuclear membrane, induce an increase in the expression of lamin A/C, enhance the mechanical strength of the nucleus, and thereby inhibit the migration and invasion abilities of cancer cells.	In vitro	[307]

In the distant colonization stage, tumor cell nuclei face a specialized mechanical tumor microenvironment that gradually transitions from vascular structural resistance to organ‐specific solid tissue constraints. The nucleus precisely senses and transduces this mechanical input through multilayered response mechanisms. At the mechanical response level, the nucleus modulates its stiffness and morphology to navigate through narrow vascular gaps. Additionally, in response to varying substrate stiffness in different target organs, the nucleus undergoes adaptive remodeling. In soft tissues, such as the brain, nuclear lamina protein expression is downregulated to soften the nucleus, while in hard tissues like bone, lamina protein expression is upregulated to enhance nuclear stiffness. This enables the nucleus to strike an organ‐specific balance between “plasticity” and “stability.” At the mechanical transduction level, mechanical memory, activated by organ‐specific stiffness differences, drives organ‐specific colonization programs through chromatin remodeling and transcriptional reprogramming. This nuclear mechanobiological network allows tumor cells to adapt to the mechanical environment of distant organs, integrating mechanical memory and initiating organ‐specific colonization programs while maintaining nuclear structural integrity and functional plasticity, thereby laying the foundation for micro‐metastasis formation.

Moreover, during tumor metastasis, the spatiotemporal heterogeneity of physical characteristics not only determines the immediate mechanical response and mechanotransduction of the cell nucleus to mechanical stimuli but also significantly impacts the subsequent biological behavior of tumor cells. Mechanical stimuli from previous stages can persistently influence the cell's behavior and nuclear function in the next phase through mechanisms such as nuclear mechanosignaling, cytoskeletal/chromatin remodeling, epigenetic adjustments, and the formation of “mechanical memory.”

During primary detachment, tumor cells are exposed to elevated ECM stiffness, solid pressure, and stromal fluid stress. These mechanical inputs are transmitted to the nucleus, triggering NE and cytoskeleton remodeling, nuclear translocation of mechanosensitive transcription factors like YAP/TAZ, epigenetic modifications, and alterations in chromatin accessibility [11, 269, 270]. These nuclear mechanical adaptations do not immediately fade after tumor cell detachment. Instead, they are retained as “mechanical memory” during cell migration, allowing tumor cells to maintain invasion‐ and stress‐related transcriptional programs upon entering the bloodstream [27]. In primary tumors, increased matrix stiffness, modeled using hydrogels with adjustable rigidity, has been shown to influence gene expression in oral squamous cell carcinoma cells. Under high stiffness conditions, mechanical signals modulate the tumor cell gene expression profile, conferring a survival advantage in circulation. This is manifested by significantly enhanced survival rates, upregulation of anti‐apoptotic protein Bcl‐2, and EMT‐related N‐cadherin, while pro‐apoptotic BAX and epithelial marker E‐cadherin are downregulated. Concurrently, the PI3K/AKT pathway is notably activated [271]. In vitro experiments simulating the circulatory system further show that breast CTCs with these EMT and anti‐apoptotic profiles (Vimentin, N‐cadherin, Bcl‐2 upregulation; E‐cadherin, EpCAM, and BAX downregulation) exhibit enhanced survival under simulated blood flow shear stress (20 dyne/cm^2^) [103]. During circulatory survival, tumor cells face high‐speed, transient mechanical events like fluid shear and microcapillary radial compression. While most CTCs die, the surviving cells exhibit superior mechanical adaptation, including higher nuclear repair capacity, more stable chromatin orientation, and sustained YAP/TAZ activation, which allow them to survive in the complex mechanical and biochemical microenvironment of distant sites [102, 272, 273]. Notably, the 2023 ASCO report indicates that in early‐stage breast cancer patients, the mechanical conditioning score (MeCo score) of CTCs is significantly higher than that of primary tumors. Furthermore, the MeCo score in metastatic lesions surpasses that of CTCs. An elevated MeCo score reflects enhanced nuclear mechanoadaptation and mechanical memory in tumor cells, facilitating their survival and metastatic potential during circulation and colonization. This increase in MeCo score correlates with poorer clinical prognosis [239, 274, 275]. Thus, there exists clear “mechanical continuity” across the three metastatic stages. Mechanical stimuli from previous stages persist through nuclear mechanosensing, chromatin remodeling, and epigenetic markers, continuously influencing cellular fate and nuclear transcriptional state in the subsequent stages. This significantly enhances the likelihood of successful metastasis.

### 3.4 Dual‐Dancer in a Mechanical World: Functional Differences of YAP and TAZ and the Path to Nuclear Stability

#### 3.4.1 Different Roles of YAP and TAZ in Mechanical Activation and Tumor Metastasis

In traditional studies, YAP and TAZ have often been regarded as functionally redundant transcriptional co‐activators due to their co‐activation in the Hippo pathway. However, recent research has revealed their distinct roles in activation patterns and regulation of tumor metastasis [276, 277, 278]. First, their mechanical activation mechanisms may differ. Molina et al. found that, in a 3D electrospun scaffold model of osteosarcoma, both YAP and TAZ exhibited enhanced nuclear localization as matrix stiffness decreased. However, in the stiffness‐variable 3D environment, YAP showed greater sensitivity in nuclear accumulation, while TAZ expression and localization remained relatively stable, suggesting differential mechanisms in stiffness sensing between the two [279]. Second, YAP and TAZ nuclear localization may vary across different cancer types. While studies in most cancers confirm co‐localization of YAP and TAZ as transcriptional factors, Wang et al. observed that, in hepatocellular carcinoma, about 70% of cases showed nuclear activation of either YAP or TAZ alone, rather than both, indicating subtype‐specific differences in their activation patterns in clinical hepatocellular carcinoma [276]. Furthermore, YAP and TAZ may exhibit different roles in regulating cellular behaviors. In non‐small cell lung cancer, Shreberk‐Shaked et al. found, through genomic and transcriptomic analyses, that YAP tends to regulate genes related to cell division and the cell cycle, while TAZ more significantly affects genes involved in ECM remodeling and cell migration, highlighting their distinct roles in metastasis‐related pathways [280]. Taken together, although YAP and TAZ are often co‐upregulated in various cancers, their distinct sensitivities to mechanical stimuli, downstream target gene profiles, and regulatory pathways lead to divergent roles in tumor metastasis. This functional divergence suggests that, when discussing the mechanisms of mechanical‐induced tumor metastasis, it is crucial to differentiate between YAP and TAZ. Such distinctions may help refine mechanistic pathways and offer more precise strategies for targeted intervention.

#### 3.4.2 Balance Between Genomic Stability and Gene Expression in Cell Nuclei Under Mechanical Stimulation

In Section 3.1.2, we previously discussed how mechanical stimulation, while promoting tumor cell migration through nuclear lamina softening, can undermine nuclear membrane stability when excessive [11, 51, 122, 150]. This destabilization can lead to DNA damage [26]. In this context, chromatin can respond to mechanical stress through epigenetic modifications, such as the reorganization or accumulation of heterochromatin markers, thereby increasing nuclear stiffness to better accommodate external mechanical forces [24]. However, the dense structure of this chromatin may, to some extent, suppress overall transcriptional activity. Consequently, a critical question arises: how do cells maintain a balance between genomic stability and gene expression? This balance likely reflects the complex mechanisms by which the nucleus responds to mechanical signals. Current research suggests that this balance may be explained through both temporal and spatial dimensions. From a temporal perspective, chromatin may exist in a relatively decondensed state to rapidly adapt to external stress in the short term, but under sustained pressure, it tends to compact, achieving a balance between short‐term flexibility and long‐term stability [24]. From a spatial perspective, chromatin also exhibits heterogeneity: mechanisms like phase separation enable chromatin to simultaneously maintain “compact” and “loose” states within the same nucleus. This allows for the overall stability of the genome while facilitating the rapid activation of specific gene regions. As a result, even in environments where overall chromatin condensation is dominant, genes closely associated with metastasis can still maintain high transcriptional activity, reflecting the tumor cell's ability to prioritize the expression of oncogenes [164, 167, 281, 282].

In summary, during the early stages of metastasis, tumor cells achieve nuclear softening through the remodeling of nuclear lamina and related structures, while preserving genomic stability via chromatin condensation. This results in a unique “soft on the outside, rigid on the inside” nuclear mechanical characteristic. This synergistic regulation not only ensures the nuclear deformability required for crossing physical barriers but also, through heterochromatin remodeling, prevents genomic instability caused by NER. Notably, even in high‐stiffness microenvironments where chromatin generally undergoes compaction, tumor cells can dynamically and locally modulate chromatin to maintain high transcriptional activity of genes critical to metastasis. This ability to balance deformability, genomic stability, and efficient expression of key oncogenes under extreme mechanical pressure provides an essential molecular and functional foundation for the progression of metastatic cascades.

## 4 Clinical Strategies for Targeting Cell Nuclei in Treating Cancer Metastasis (Table 3)

### 4.1 Targeted Modulation of Sensitive Structures in Nuclear Mechanics

In therapeutic strategies targeting tumor metastasis, the nucleus, specifically its mechanosensitive structures, has emerged as a promising focus of investigation. As previously discussed, elevated matrix stiffness and shear stress can induce nuclear deformation, membrane rupture, conformational changes in NPCs, and nuclear lamina remodeling. These mechanical perturbations disrupt the chromatin architecture, activate signaling pathways, and reprogram gene expression profiles, collectively enhancing the migratory and metastatic potential of tumor cells. Targeted pharmacological interventions aimed at reinforcing nuclear mechanical integrity, such as stabilizing the NE, restoring nuclear pore barrier function, or upregulating key lamina components such as lamin A/C, have shown potential for increasing nuclear stiffness and reducing nuclear deformability. These approaches offer innovative avenues for suppressing metastasis by limiting cellular plasticity and invasiveness from the perspective of nuclear mechanics.

#### 4.1.1 Lamin A/C

Many cancer cells reduce nuclear mechanical stiffness by downregulating or reorganizing lamin A/C proteins, thereby facilitating their passage through a confined mechanical microenvironment [120, 121]. Consequently, restoring or enhancing the expression of lamin A/C can increase the nuclear stiffness of tumor cells, limiting their physical deformation and migratory capacity, which is an important strategy for cancer treatment. Studies across various cancer models, including breast, cervical, and pancreatic cancers, have investigated the regulation of lamin proteins to inhibit tumor proliferation and metastasis [283, 285, 286]. This can be achieved through methods such as inhibiting the farnesylation of prelamin A or upregulating the LMNA gene, which enhances the accumulation of lamin A/C in the nucleus, significantly increasing nuclear stiffness and reducing the migration and invasion of cells in three‐dimensional matrices. Moreover, lamin A/C plays a central role in maintaining nuclear stiffness and genomic stability. Excessive inhibition of lamin A/C expression may compromise nuclear stability, and potentially enhance cell migration and invasion in three‐dimensional matrices [283, 287]. In conclusion, regulating the expression and function of the nuclear lamina protein, lamin A/C, has emerged as a promising avenue for inhibiting tumor metastasis. However, given that lamin A/C is involved in multiple cellular functions, such as structural stability, gene expression regulation, and cell differentiation, alterations in its expression could disrupt normal cell function and increase the risk of side effects [308]. Therefore, future research must explore the role of lamin A/C in tumor biology and develop more precise and effective regulatory strategies to advance the clinical application of such therapies.

#### 4.1.2 LINC Complex

The LINC complex mediates the transmission of cytoskeletal tension to the NE and acts as a critical hub for transducing mechanical signals into the nucleus [43, 48]. Theoretically, targeting key components of the LINC complex, such as SUN1/2 and Nesprins, can diminish the responsiveness of the nucleus to external mechanical stress, thereby attenuating EMT and metastatic potential. This concept has attracted significant research interest in recent years. For example, the bis‐Schiff base salt compound M2 stabilizes G‐quadruplex structures in the promoter region of LINC00273, suppressing its expression and markedly impairing tumor cell migration and invasion [287]. Although no clinically approved drugs currently target SUN1/2 or Nesprins directly, several strategies have been explored to disrupt LINC function, including interference with SUN‐KASH domain interactions and modulation of SUN1/2 disulfide bonds using protein disulfide isomerase (PDI) inhibitors [309]. These approaches demonstrate the potential of modulating LINC structure or function to influence tumor cell motility and invasion. Future research should focus on developing specific molecules that regulate the assembly or stability of the LINC complex, offering novel targets for anti‐metastatic therapies.

#### 4.1.3 Nuclear Membrane and NPCs

In the TME, mechanical stimuli, such as increased matrix stiffness and shear stress, can induce local nuclear membrane rupture, leading to DNA damage [26, 34]. ATR kinase plays a critical role not only in DNA damage repair but also in maintaining nuclear structural integrity. As a result, ATR inhibitors are potential therapeutic agents for inhibiting tumor metastasis. Berzosertib (VE‐822), the first highly selective ATR inhibitor to enter clinical trials, has demonstrated antitumor activity against various cancer types [302]. Recently, Camonsertib has been shown to have similar effects [301]. These findings suggest that ATR‐targeted inhibitors may effectively suppress tumor metastasis by disrupting DNA damage repair and preserving nuclear stability.

### 4.2 Physiotherapy

**FIGURE 6:**
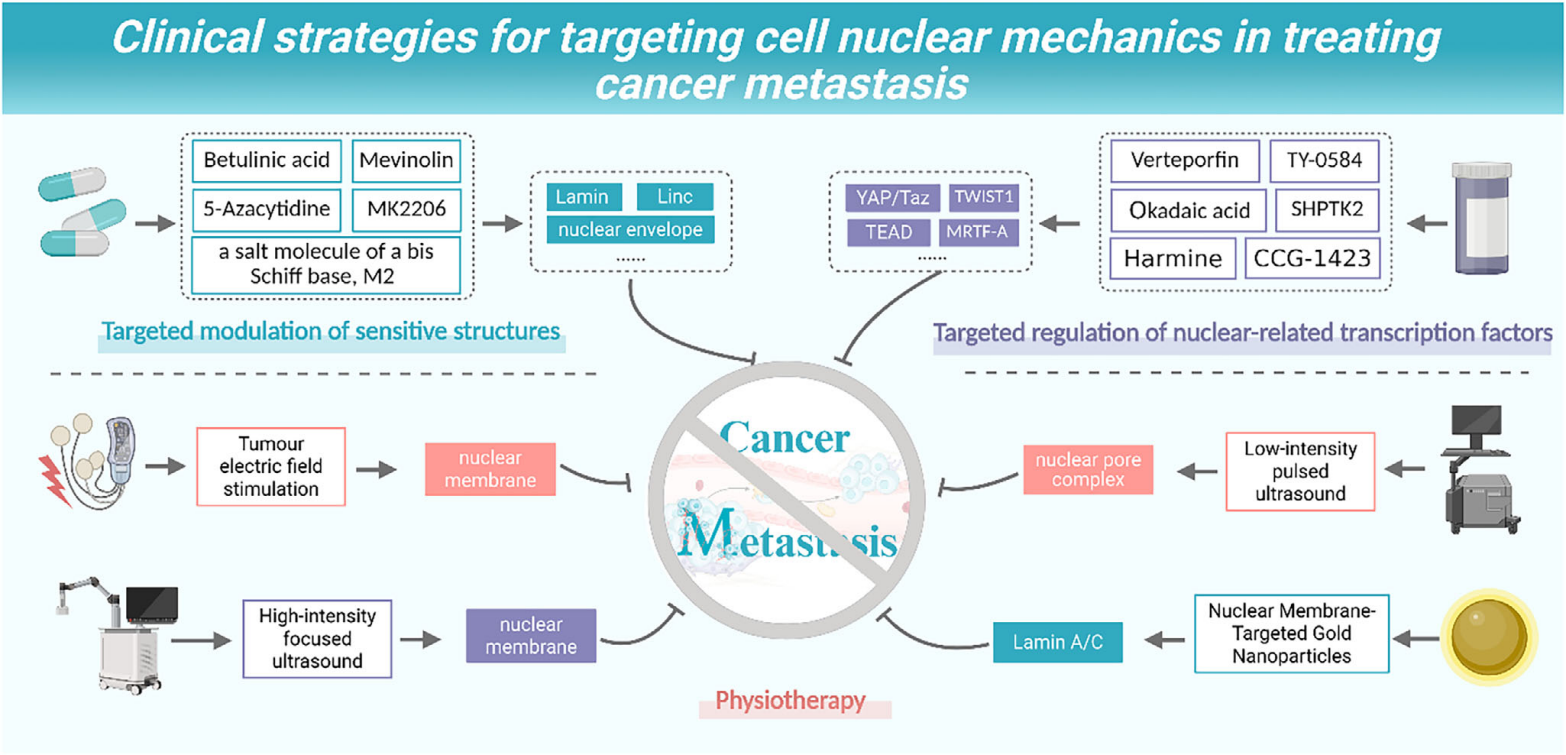
Clinical strategies targeting nuclear in cancer metastasis therapy. Targeting nuclear mechanical structures and their associated mechanisms offers diverse therapeutic strategies for inhibiting tumor metastasis. First, modulation of nuclear envelope components, such as lamins (e.g., lamina proteins A/C(Lamin A/C)) and the linker of nucleoskeleton and cytoskeleton(LINC) complex, can limit tumor cell deformation and migratory capacity. Second, targeting nuclear transcription factors such as myocardin‐related transcription factor A(MRTF‐A), yes1 associated transcriptional regulator(YAP)/transcriptional coactivator with PDZ‐binding motif(TAZ)‐TEA domain family member 1(TEAD), and twist‐related protein 1(TWIST1) can suppress tumor cell migration and invasion. Additionally, physical therapies, including high‐frequency low‐intensity pulsed ultrasound and shockwave therapy, can regulate the mechanical state of cancer cell nuclei, inducing DNA damage and apoptosis, thereby reducing metastatic potential. These strategies not only provide novel approaches to targeting cancer metastasis but also offer advantages such as being noninvasive or minimally invasive, highly targeted, and with reduced side effects, paving the way for precision cancer therapy.

In recent years, physical therapy, specifically mechanical therapy, has emerged as a promising tumor treatment strategy targeting nuclear mechanics. This approach regulates the mechanical properties of the cancer cell nuclei using external mechanical forces, thereby inhibiting their metastatic potential. Studies have shown that pulsed ultrasound can induce NER in cancer cells, leading to DNA damage and activation of the cGAS‐STING signaling pathway. This promotes apoptosis and triggers an immune response, thereby inhibiting tumor proliferation and metastasis. In contrast, tumor‐targeting electric field stimulation also causes localized NER and perforation, but through a different mechanism. This leads to micro‐nucleation and activation of Caspase‐1, which cleaves GSDMD and further exacerbates DNA damage, triggering pyroptosis and inhibiting the migration and invasion of cancer cells. [303]. Additionally, in the HEYA8 cell model in vitro, gold nanoparticles (AuNPs) that accumulate at the nuclear membrane operate through two primary mechanisms to inhibit cancer cell migration and invasion: first, they directly enhance the mechanical rigidity of the NE; second, they stimulate the overexpression of the nuclear lamina protein Lamin A/C. The combined effect significantly increases nuclear stiffness, thereby suppressing the cells' migratory and invasive capabilities [307]. These mechanical therapies do not rely on traditional chemotherapy or radiotherapy, are noninvasive or minimally invasive, and highly precise with minimal side effects, which provides a novel approach for interfering with tumor mechanical signals and inhibiting metastasis. With a deeper understanding of nuclear mechanics and regulatory mechanisms, future advancements may involve combining mechanical and molecular pathways, such as Piezo1/YAP, to develop more precise physical therapy regimens. These could further enhance the anti‐metastatic efficacy and clinical application potential. (Figure 6)

### 4.3 Targeted Regulation of Nuclear‐Related Transcription Factors

In the context of mechanical signal transduction, the focus of upstream mechanical stimuli is on the activity and localization of nuclear transcription factors [11]. Small molecule interventions targeting these key nodes have demonstrated significant anti‐metastatic potential across various tumor models. First, MRTF‐A, a downstream effector of the RhoA/ROCK pathway, plays a crucial role in driving tumor cell migration and invasion through its nuclear localization. Treatment with the small molecule CCG‐203971 and its analogues in a melanoma mouse model notably reduced the number and volume of lung metastases and inhibited cell migration and invasion in vitro [298, 299, 300]. Second, the Hippo pathway effectors YAP/TAZ convert mechanical signals into gene expression by binding to the TEAD family of transcription factors. Their accumulation in the nucleus is essential for promoting EMT and metastasis. Verteporfin specifically disrupts the YAP–TEAD interaction, forcing YAP to remain in the cytoplasm, where it is degraded. This action downregulates EMT‐associated genes such as CTGF, CYR61, and SNAIL. In breast cancer and pancreatic ductal adenocarcinoma models, verteporfin treatment significantly reduced distant metastases [289, 310]. These findings highlight its dual role in selectively inhibiting metastasis and primary tumor growth. Additionally, inhibitors such as CA3, which covalently bind to palmitoylation sites on TEAD, could suppress primary tumor growth and reduce distant metastasis in osteosarcoma and mesothelioma models [293, 294, 295]. TWIST1, a master regulator of EMT, relies on heterodimer formation with the E2A protein for its nuclear activity and protein stability. Harmine promotes the degradation of TWIST1–E2A heterodimers, significantly inducing senescence or apoptosis in NSCLC and breast cancer cells. This also led to reduced lung metastasis and prolonged survival in epidermal growth factor receptor (EGFR)‐ and Kirsten rat sarcoma virus (KRAS)‐driven mouse xenograft models [297].

It is important to note that while transcriptional regulators such as YAP/TAZ, Twist1, and MRTF‐A play key roles in nuclear mechanosignaling, they are not solely controlled by mechanical stimuli. Rather, they function as “mechanical‐biochemical integrators,” decoding multiple upstream signaling pathways. For instance, YAP/TAZ are highly sensitive to ECM stiffness and actin–myosin tension, while also integrating signals from the Hippo, G protein‐coupled receptor, and Wnt pathways [123, 152, 311]. Twist1 is regulated by TGF‐β and other growth factors during EMT, and MRTF‐A's nuclear translocation, although dependent on mechanosensitive actin polymerization, can also be activated by serum and other biochemical stimuli [15, 312, 313, 314, 315]. These examples demonstrate that the mechanosensitivity of transcription factors often overlaps with biochemical regulation. This signal crosstalk limits the applicability of strategies targeting individual transcription factors, such as YAP, Twist1, or MRTF‐A, in mechanobiology‐driven interventions.

While no drug currently exists that can completely block mechanical stimulus‐driven nuclear translocation without affecting biochemical activation, recent experimental studies have provided new insights. For example, it has been shown that under mechanical stimulation, YAP and Importin β exhibit coordinated spatiotemporal distribution changes. Inhibition of Importin β significantly disrupts the nuclear localization of YAP under mechanical conditions, suggesting that the nuclear import process may represent a manipulable upstream node in mechanosignaling [316]. Furthermore, intervening at nodes closer to the source of mechanosensation, such as the LINC complex, may more effectively and specifically block the transmission of mechanical signals to downstream transcription factor nuclear translocation, providing a selective window for “mechanical interventions [74].” While these strategies cannot achieve absolute mechanospecificity, they offer a novel direction for interventions that distinguish mechanical signals from traditional biochemical pathways, providing potential avenues for future mechanobiology‐driven drug development.

Finally, it should be noted that most nuclear mechanics‐related agents currently remain at the stage of in vitro studies or animal models, and no drug has yet entered late‐stage clinical trials with “direct modulation of nuclear mechanics to suppress tumor metastasis” as a defined clinical endpoint. Nevertheless, these candidates are not devoid of clinical or translational evidence. For example, the epigenetic regulator 5‐azacytidine has been approved for the treatment of myelodysplastic syndromes and has demonstrated manageable safety profiles and antitumor activity in multiple phase II/III oncology trials (NCT01566695, NCT03703375). MK‐2206 has been evaluated in several phase I/II trials in solid tumors, establishing its safety and tolerability (NCT00848718, NCT01169649). Berzosertib (M6620) has undergone multiple phase I/II clinical evaluations in combination regimens, leading to the determination of recommended dosing (NCT02157792, NCT03641313). In addition, Verteporfin, clinically used for photodynamic therapy in retinal diseases, is being explored for its potential to inhibit mechanotransduction‐related pathways and thereby modulate tumor growth and invasion (NCT04590664, NCT03033225). Collectively, these data indicate that these agents possess favorable pharmacokinetic and safety profiles and have shown antitumor efficacy in specific oncological contexts.

At the same time, with the rapid advances in tumor mechanobiology and nuclear mechanics, this field is gaining increasing attention and translational momentum. Mechanistic insights from nuclear mechanobiology are providing a conceptual framework for the development of novel diagnostic and therapeutic strategies. Looking ahead, continued progress in the quantification of mechanical biomarkers, in vivo mechanical feedback imaging, and multi‐omics data integration is expected to enable the rational design of clinical trials targeting nuclear mechanics, thereby facilitating the translation of these emerging strategies into effective anti‐metastatic interventions.

## 5 Discussion

As the central sensor and decoder of mechanical signals in the tumor metastasis cascade, the dynamic response mechanism of the nucleus offers a novel mechanical perspective for understanding the multistep metastasis process. This review systematically examines the mechanical perception and transduction pathways as well as the remodeling effects on key signaling pathways (such as YAP/TAZ‐TEAD, MRTF‐A‐SRF, and cGAS‐STING), along with the epigenetic landscape of the nucleus throughout the entire process, from tumor cell detachment at the primary site, survival in circulation, and colonization in distant organs.

Although the role of mechanical signals in tumor metastasis on the cell nucleus has been identified, this field still faces the following key challenges and core issues that require in‐depth exploration:

1. Mechanisms of Intercellular Transmission of Mechanical Memory Remain Elusive: The nucleus can establish “mechanical memory” under sustained mechanical stimulation, maintaining a pro‐metastatic phenotype. However, it remains unknown whether and how such mechanically induced memory is transmitted between cells. For instance, could exosomes secreted by tumor cells in stiff microenvironments—carrying specific membrane proteins, miRNAs, DNA fragments, or other cargo—act as carriers of mechanical information, mediating the spread of mechanical memory across cell populations? Elucidating this mechanism is crucial for understanding the collective behavior of metastatic lesions.
2. Disconnect Between Mechanosensing Studies and Intranuclear Response Mechanisms: Current research predominantly focuses on extracellular mechanical signal perception and intracellular signal transduction, while understanding of the “terminal” events—such as how signals entering the nucleus drive phenotypic transformation through chromatin remodeling, alterations in nuclear mechanical properties, and transcriptional reprogramming—remains inadequate. At the same time, scholars specializing in intranuclear structure and function, despite making advances in chromatin modification and nuclear mechanics, often fail to causally link these phenomena to the dynamic processes of cancer metastasis, such as EMT, CTC formation, and colonization. This has led to a fragmented research landscape described as “pathway studies stopping at the NE, and nuclear studies detached from the metastatic context.” Such division makes it difficult to systematically establish a complete, continuous evidence chain of “mechanical stimulation – signal transduction – nuclear response – phenotypic shift.” Relying on multiple independent studies to bridge these gaps significantly weakens both the strength of evidence and causal credibility. To overcome this limitation, future work must deeply integrate tumor biology, nuclear biomechanics, and epigenetics within unified models to clarify how mechanical signals precisely regulate chromatin states and nuclear physical properties upon entering the nucleus, thereby driving metastatic adaptation. This is not only key to understanding metastasis in its entirety but will also provide a more solid theoretical foundation for developing targeted intervention strategies.
3. Time‐Dependent Heterogeneity of Mechanical Signals and Its Role in Nuclear Mechanics Remains Underexplored: Although we have developed some understanding of the spatiotemporal heterogeneity across different stages of the metastatic cascade, research into temporal heterogeneity has largely been confined to the sequential framework of tumor progression due to methodological limitations. The dynamic changes of mechanical signals over time and their biological significance remain insufficiently explored. Nevertheless, emerging evidence indicates that the time‐dependent fluctuations of mechanical signals significantly influence tumor progression. For example, the observed nocturnal acceleration of breast cancer metastasis suggests that hemodynamic parameters may exhibit circadian oscillations, thereby regulating the fate of CTCs. Furthermore, at the single‐cell level, the mechanical properties of the nucleus also display significant temporal heterogeneity. Cell cycle‐dependent changes in nuclear mechanics, such as nuclear softening during the S/G2 phase, may profoundly affect a cell's perception of matrix stiffness and its metastatic capability. This reveals a complex, yet unelucidated, interaction network between the cell cycle and mechanical signal perception. Future efforts must develop experimental techniques with high temporal and spatial resolution to capture the dynamic features of mechanical signals and intranuclear responses over time, thereby revealing the complete map of how spatiotemporal mechanical heterogeneity regulates metastasis.
4. The In Vitro Models Still Have Limitations in Simulating Dynamic Mechanical Environments: In tumor metastasis research, existing in vitro models have significant limitations in simulating the multiscale dynamic mechanical environment present in vivo. For instance, commonly used 2D flow chamber models for the circulatory phase fail to account for disturbances in local shear fields caused by red blood cells and other components. Meanwhile, static models based on fixed matrix stiffness cannot reflect the temporal fibrosis and stiffening evolution of the tumor ECM. In recent years, a series of more physiologically relevant experimental systems have gradually emerged, such as tumor‐transendothelial migration chips. These systems integrate key factors—including endothelial barriers, FSS, and immune cells—under 3D conditions, significantly enhancing the physiological relevance of modeling the circulatory extravasation phase. In terms of matrix mechanics, studies have begun employing models with stiffness gradients, regional heterogeneity, or dynamically tunable ECM to compensate for the shortcomings of traditional static models. However, these models still have limitations in simulating the dynamic and complex nature of the in vivo physiological environment. Therefore, future work must further integrate 3D dynamic culture systems, organ‐on‐a‐chip technology, complex fluidic systems, and high‐resolution live imaging techniques. This will enable the systematic analysis of mechanical signal transmission and its intranuclear response under conditions that more closely approximate physiological reality.

Several unresolved issues still require further investigation, such as the distinct roles of YAP and TAZ, the context‐dependent functions of lamin A/C, and the impact of nuclear softening/stiffening on tumor metastasis. Although we have incorporated recent research into the main text discussing these topics, we acknowledge that direct studies conclusively addressing these questions are still lacking. We believe that deeper exploration into the differences and interactions among these mechanisms will not only enhance our understanding of the complexity of tumor metastasis but also provide more precise strategies for future targeted interventions.

At the level of therapeutic strategies targeting mechanical intervention, the following points warrant attention:

1. Windowing of Intervention Timing: Existing evidence confirms that the sustained action of mechanical signals and the accumulation of mechanical memory accompany the progressive accumulation and evolution of malignant phenotypes, such as immune evasion. Early intervention in mechanotransduction pathways may effectively delay metastasis initiation. In contrast, for established late‐stage metastatic lesions, there is a greater need to co‐target mechanically driven pathways (such as nuclear mechanical responses) with other treatment modalities like immunomodulation to overcome accompanying mechanics‐dependent immunosuppression. Therefore, precisely determining the optimal intervention window is critical for therapeutic success.
2. Synergistic Targeting of Mechanical and Genetic Defects: Tumor cells with specific genetic defects (such as NE protein mutations, DNA repair deficiencies) may exhibit abnormal sensitivity or vulnerability to mechanical stimuli, presenting a potential “Achilles' heel” for cancer therapy. Exploring how mechanical stress exacerbates survival pressure or genomic instability in genetically defective cells, and comprehensively considering both mechanical and genetic factors, may facilitate the development of precision synergistic strategies.
3. Integration of Multi‐Scale Technologies: Although targeting nuclear mechanics has a clear biological rationale and potential therapeutic value in inhibiting invasion and metastasis, no clinical trials have yet been established with “preventing metastasis via nuclear mechanics regulation” as a primary endpoint. The reasons for this are likely multifaceted. On one hand, the clinical biomarker system for nuclear mechanics as a therapeutic target is not yet mature, lacking quantifiable evaluation metrics (such as nuclear deformability, nuclear stress transmission, and mechano‐sensitive transcription) needed to define trial endpoints. On the other hand, small molecules or inhibitors targeting the nuclear skeleton or LINC complex face pharmacokinetic challenges such as inefficient nuclear membrane penetration and limited targeting specificity. Physical methods like focused ultrasound or electric field stimulation encounter difficulties in precisely locating and intervening in deep‐seated metastases.

Furthermore, excessive modulation of nuclear stiffness may carry the potential risk of inducing genomic instability, making clinical advancement more cautious. Therefore, to promote the clinical translation of nuclear mechanics‐targeting strategies, a “multi‐scale integrated” research and development framework is required. This should include leveraging nanotechnology to enhance nuclear delivery efficiency, utilizing biomaterials to construct controllable mechanical microenvironments, employing advanced imaging to optimize the spatial and temporal precision of physical interventions, and combining these with existing molecular‐targeted or immunotherapeutic strategies to achieve synergistic effects. Such a cross‐disciplinary integration of physics, biology, and engineering will not only help overcome current bottlenecks but also aid in forming clinically actionable intervention paradigms, laying the groundwork for the future translation of anti‐metastasis strategies based on nuclear mechanics.

## Funding

This work was supported by the National Natural Science Foundation of China (82430123), the Noncommunicable Chronic Diseases‐National Science and Technology Major Project (2024ZD0521400), the Shandong Taishan Scholars Specially Invited Expert Talent Project (tstp20221166), Shandong Second Medical University Scientific Research Innovation Programme (02199101), National Administration of Traditional Chinese Medicine High‐Level Key Discipline Construction Project of Traditional Chinese Medicine (ZYYZDXK‐2023125), Shandong University of Traditional Chinese Medicine Postgraduate Quality Enhancement and Innovation Project (YJSTZCX2025182).

## Conflicts of Interest

The authors declare no conflicts of interest.

## Ethics Statement

The authors have nothing to report.

## Consent

All authors consent to publication.

## Supporting information




**Supporting File**: advs74991‐sup‐0001‐SuppMat.doc.

## Data Availability

No datasets were generated or analysed during the current study.
