# Supplementary material for: Nuclear Mechanotransduction Across the Metastatic Cascade: Decoding Spatiotemporal Heterogeneity in Cancer Dissemination
Source: Adv Sci (Weinh). 2026 Apr 2;13(25):e23974. doi: 10.1002/advs.202523974 (PMC13137818; doi:10.1002/advs.202523974)
Supplement: Supplementary file 1 — Supporting File: advs74991‐sup‐0001‐SuppMat.doc. [file ADVS-13-e23974-s001.doc]

Supporting Information

Nuclear Mechanotransduction Across the Metastatic Cascade: Decoding Spatiotemporal Heterogeneity in Cancer Dissemination

Linqi Song, Jingyang Liu, Xue Wang, Minpu Zhang, and Changgang Sun*


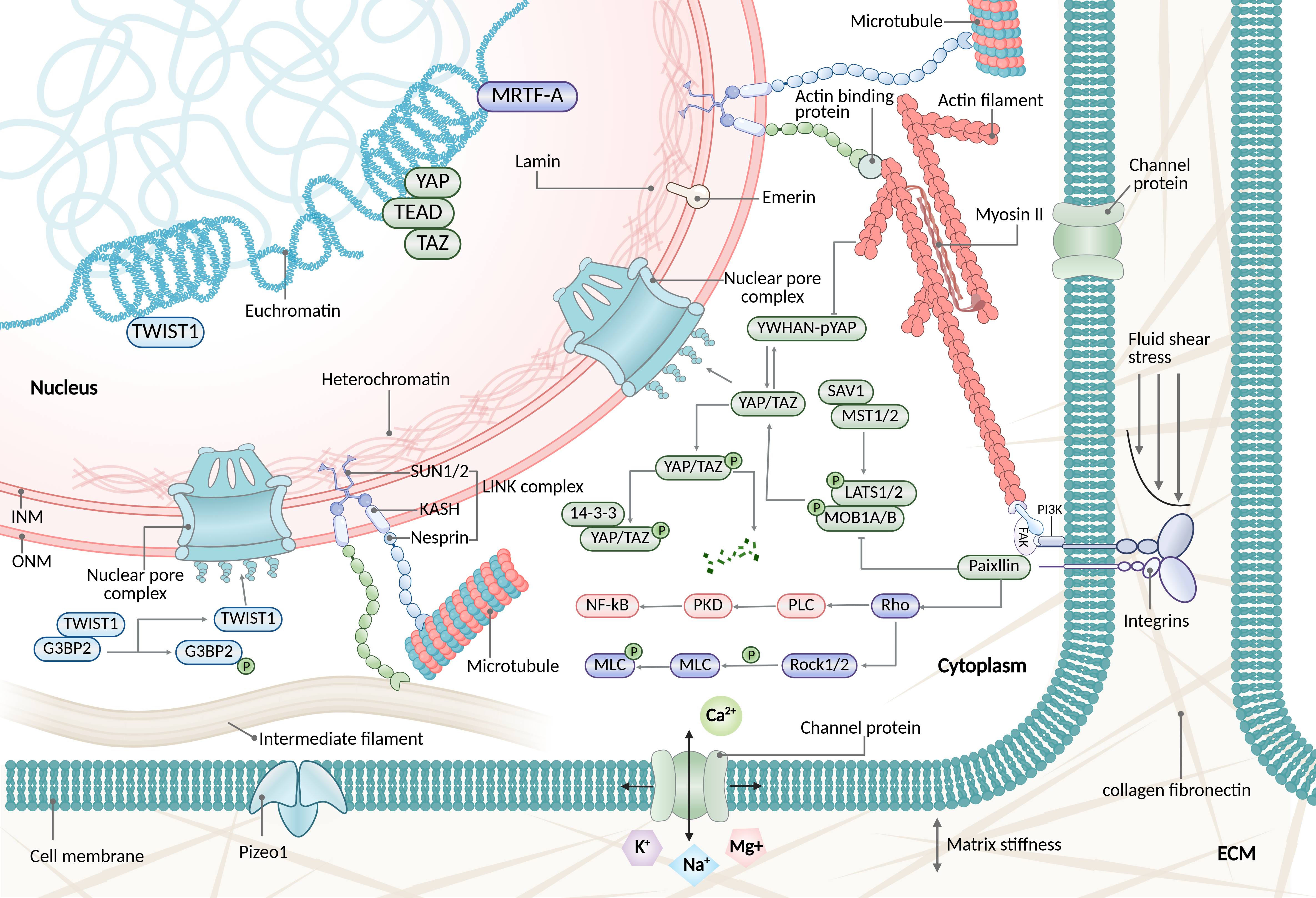


**Figure 1.** Structural Basis of Nuclear Mechanosensing and Signal Transduction. The nuclear envelope (NE), comprising the outer nuclear membrane (ONM) and inner nuclear membrane (INM), serves as the first barrier for mechanical signal conversion. Its phospholipid bilayer dynamically adjusts membrane tension in response to mechanical cues. Nuclear pore complexes, embedded within the NE, undergo conformational changes under mechanical stress, alleviating membrane tension while enhancing the nuclear import of mechanosensitive transcription factors, thereby directly coupling mechanical stimuli to gene regulation. The nuclear lamina, a dense meshwork of A-type (lamin A/C) and B-type (lamin B1/B2) intermediate filaments underlying the INM, provides the nucleus with its greatest mechanical strength, ensuring structural integrity. Together with INM-associated proteins such as lamin B receptor (LBR) and emerin, it anchors chromatin to the nuclear periphery. The linker of nucleoskeleton and cytoskeleton (LINC) complex spans the NE and physically links the cytoskeleton to the nuclear interior: Nesprin proteins on the ONM (Nesprin-1/2 bind actin filaments; Nesprin-3, intermediate filaments; Nesprin-1/2/4, microtubules) connect to Sad1/UNC-84 (SUN)-domain proteins on the INM, enabling efficient transmission of extracellular mechanical signals to the nucleus. Intranuclear chromatin organization is also critical: peripherally localized heterochromatin provides rigidity, while centrally located euchromatin maintains structural flexibility. Their dynamic interconversion modulates nuclear mechanosensitivity and facilitates adaptive transcriptional responses.


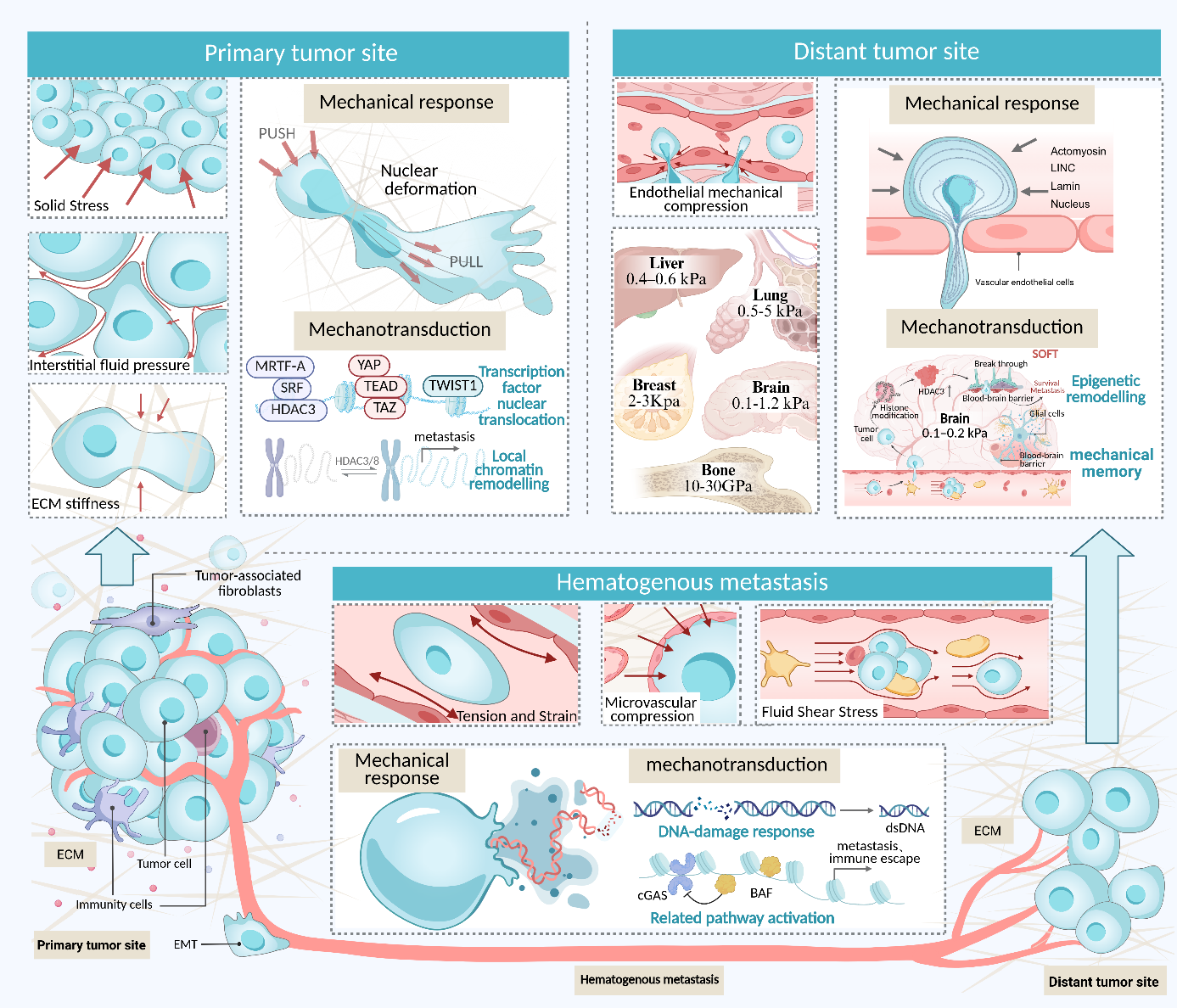


**Figure 2.** Nuclear Mechanical Response and Transduction in the Tumor Metastatic Cascade. The metastatic cascade in tumors can be divided into three distinct stages, based on differences in mechanical signals and the survival microenvironment: primary detachment, circulatory survival, and distant colonization. In the primary detachment stage, the primary mechanical challenges faced by the nucleus include increased extracellular matrix (ECM) stiffness, solid stress accumulation, and interstitial fluid pressure. During this stage, the nucleus dynamically adapts to mechanical stimuli through various responses such as softening and deformation. This leads to local chromatin remodeling, nuclear translocation of transcription factors, and activation of mechanotransduction pathways that drive invasive migration programs. In the circulatory survival stage, the main mechanical stresses are blood flow shear and compressive forces from lumen narrowing. Due to intense nuclear compression, the frequency of nuclear envelope rupture increases, triggering a mechanical response that activates repair pathways, such as the DNA-damage response and GMP-AMP synthase (cGAS)–STING. These mechanisms facilitate repair and immune evasion. In the distant colonization stage, the nucleus reshapes to adapt to the mechanical characteristics of the target organ. This transition moves from short-term passive adaptation to long-term alterations in nuclear chromatin stability. This process triggers persistent epigenetic reprogramming (mechanical memory), sustained YAP/TAZ signaling, and long-term transcriptional programs that promote proliferation and colonization.


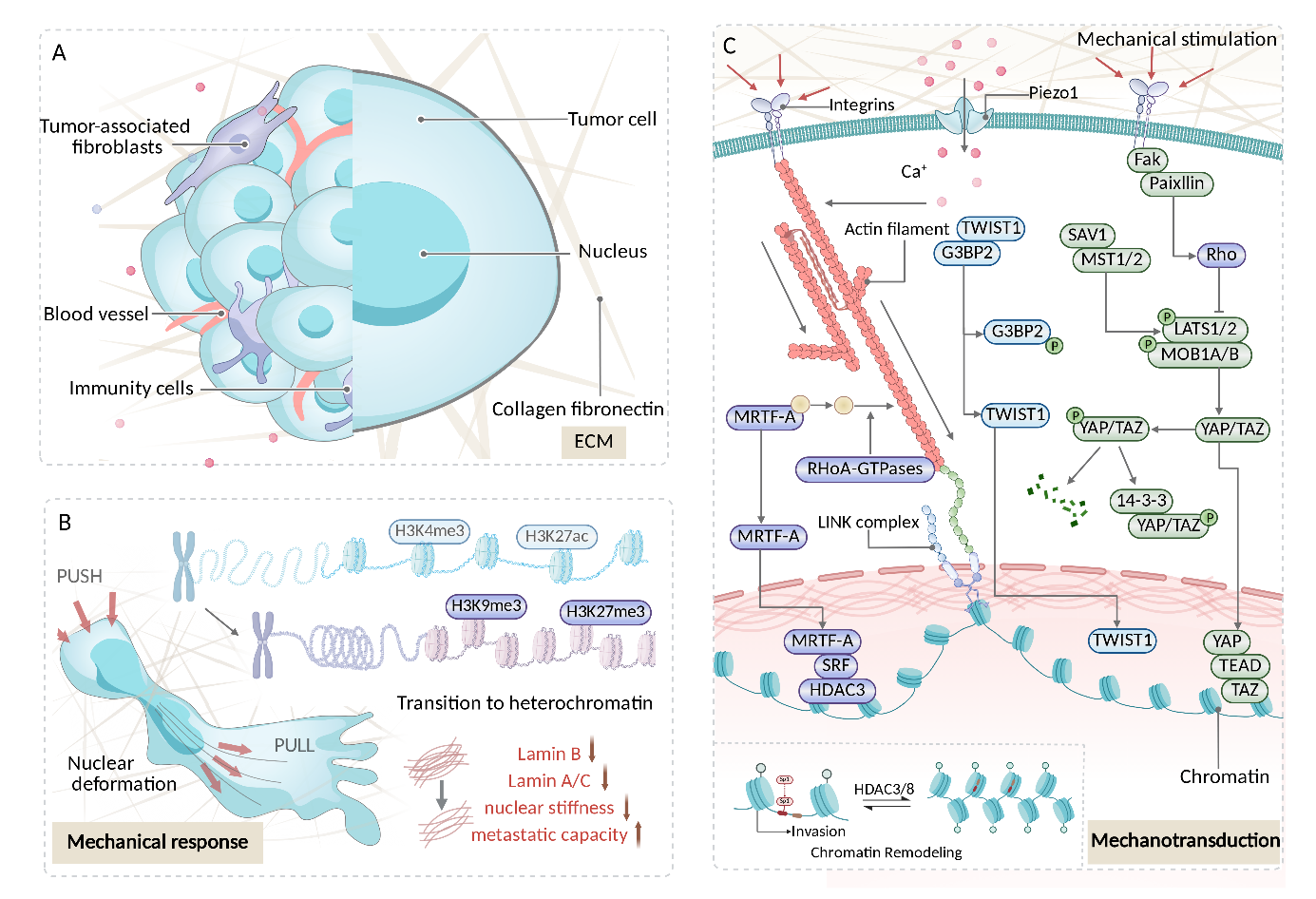


**Figure 3.** Nuclear Mechanical Responses and Signal Transduction During the Primary Detachment Stage.A. Overview of the primary detachment stage. This early metastatic phase involves three sequential steps: (1) detachment of tumor cells from the primary site, (2) invasion through the basement membrane into the surrounding extracellular matrix (ECM), and (3) intravasation into blood or lymphatic vessels. These processes are governed by mechanical cues such as ECM stiffness, solid stress, and interstitial fluid pressure. B. Nuclear mechanical responses. In response to these biomechanical stimuli, tumor cells exhibit nuclear softening—a mechanical adaptation that facilitates invasion and metastasis. This is primarily mediated by alterations in nuclear lamina composition and chromatin organization. C. Mechanotransduction mechanisms. Mechanical signals are transduced from the extracellular environment to the nucleus, where they regulate the nucleocytoplasmic shuttling of signaling molecules and induce chromatin remodeling. Key players include the TWIST1–G3BP2 complex, EPHA2/LYN-mediated phosphorylation of TWIST1, and activation of the YAP/TAZ–TEAD axis. Together with epigenetic reprogramming, these pathways reinforce epithelial–mesenchymal transition (EMT), enhance cellular detachment and invasion, and drive progression to subsequent metastatic stages.


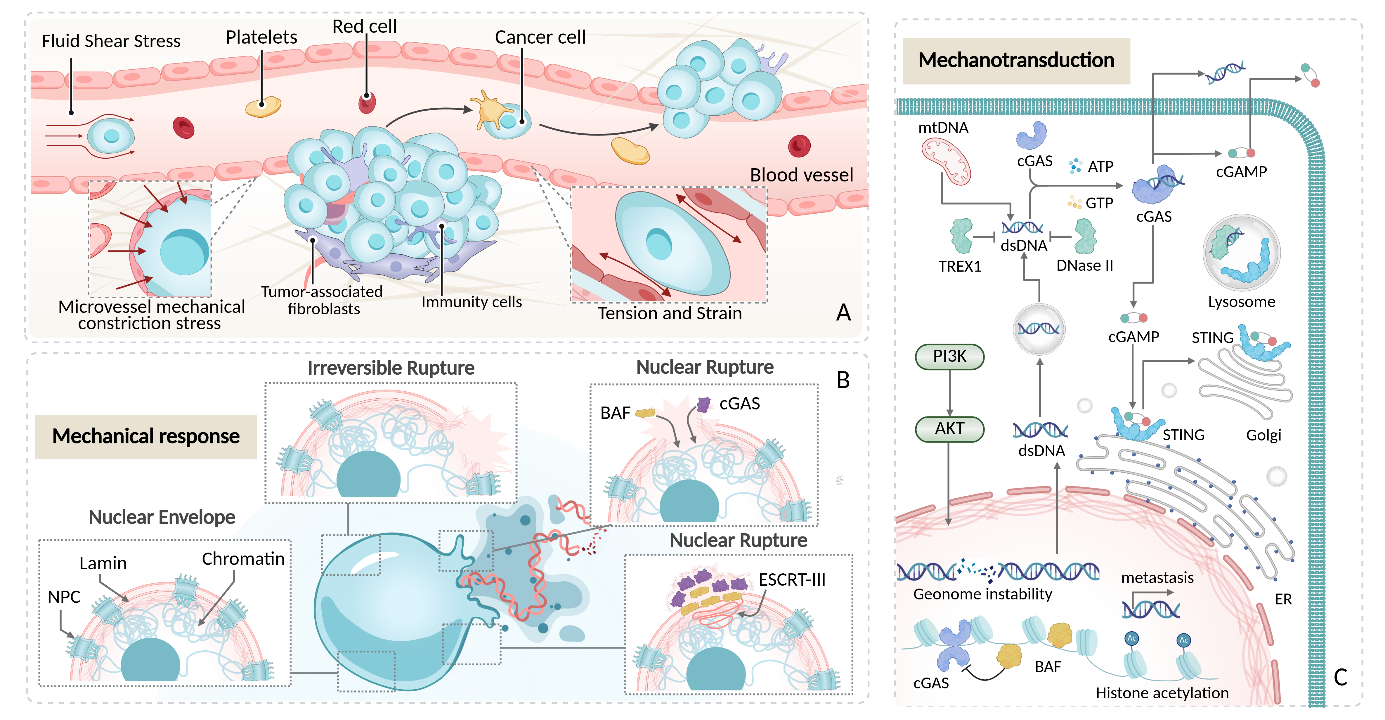


**Figure 4.** Nuclear Mechanical Responses and Signal Transduction During the Circulating Survival Stage. A. Overview of the circulating survival stage. This transitional phase occurs as tumor cells intravasate into blood or lymphatic vessels and exist as circulating tumor cells (CTCs). During this brief but critical window, cells are exposed to intense biomechanical forces—chiefly FSS from blood flow. B. Nuclear mechanical responses. In response to circulatory mechanical stress, the nucleus undergoes pronounced deformation and softening. When these stresses exceed the structural tolerance of the nuclear envelope, rupture and subsequent repair occurs. This process is orchestrated by the nuclear membrane, nuclear lamina proteins, and associated repair machinery. C. Mechanotransduction mechanisms. Within the high-shear environment of the vasculature, the nucleus activates distinct mechanical signaling cascades, including the GMP-AMP synthase (cGAS)–STING and PI3K–AKT pathways. These responses promote anti-apoptotic signaling, enhance CTC adhesion-mediated survival, and facilitate immune evasion. Concurrently, chromatin remodeling and epigenetic modifications amplify these stress-adaptive responses. Together, these nuclear adaptations significantly improve CTC viability and support their transition to the next metastatic stage.


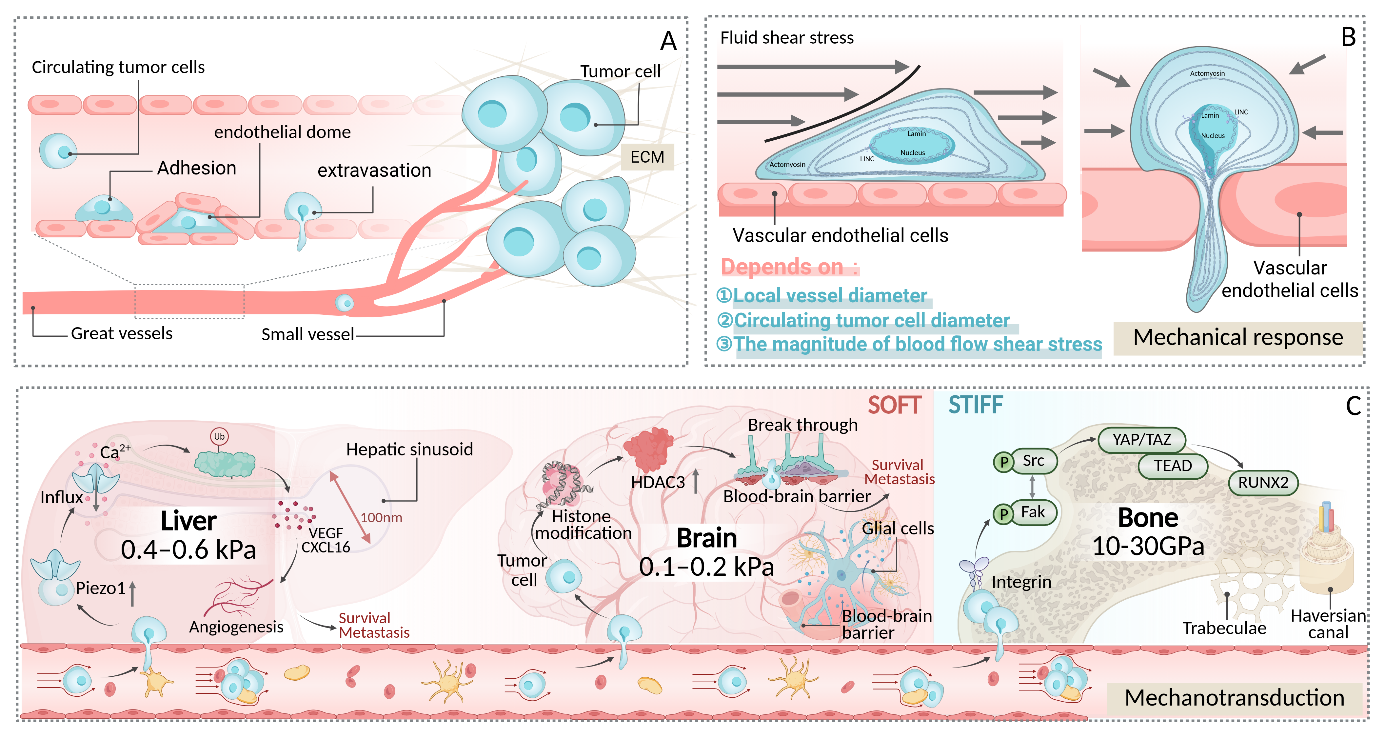


**Figure 5.** Nuclear Mechanical Responses and Signal Transduction During the Distant Colonization Stage. A. The distant colonisation phase comprises two critical steps: tumor cells traverse vascular or lymphatic barriers (extravasation) and subsequently survive and proliferate within distant tissues to form metastatic foci. This process is regulated by mechanical signals such as blood flow shear stress and stromal stiffness. B. Nuclear mechanical responses. While the types of mechanical stimuli resemble those encountered during primary detachment, the magnitude and context vary significantly across different target organs. The nucleus adapts through organ-specific activation of mechanosensitive structures, enabling successful colonization. C. Nuclear mechanotransduction. During this phase, nuclear mechanotransduction integrates organ-specific mechanical structures with "mechanical memory," adapting to the local niche through mechanisms such as YAP nuclear localization and epigenetic modifications. These processes enable tumor cells to remodel their microenvironment, establishing a positive feedback loop that supports sustained proliferation and metastatic growth.


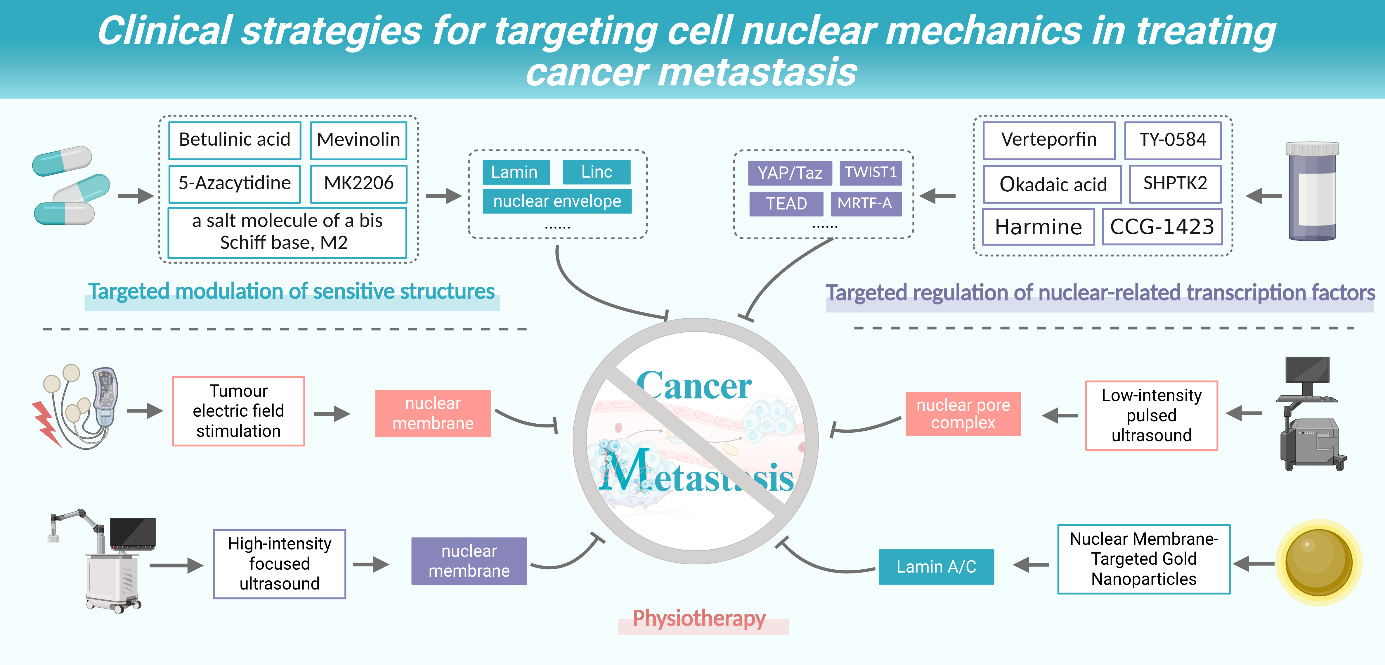


**Figure 6.** Clinical Strategies Targeting Nuclear in Cancer Metastasis Therapy. Targeting nuclear mechanical structures and their associated mechanisms offers diverse therapeutic strategies for inhibiting tumor metastasis. First, modulation of nuclear envelope components, such as lamins (e.g., Lamin A/C) and the LINC complex, can limit tumor cell deformation and migratory capacity. Second, targeting nuclear transcription factors such as MRTF-A, YAP/TAZ-TEAD, and TWIST1 can suppress tumor cell migration and invasion. Additionally, physical therapies, including high-frequency low-intensity pulsed ultrasound and shockwave therapy, can regulate the mechanical state of cancer cell nuclei, inducing DNA damage and apoptosis, thereby reducing metastatic potential. These strategies not only provide novel approaches to targeting cancer metastasis but also offer advantages such as being non-invasive or minimally invasive, highly targeted, and with reduced side effects, paving the way for precision cancer therapy.

**Table 1 The spatiotemporal heterogeneity of the mechanical tumor microenvironment during cancer metastasis**

| Table 1 The spatiotemporal heterogeneity of the mechanical tumor microenvironment during cancer metastasis | Refs | [91,91,93,95–101] | ^[6,20,101–109]^. | [110–118] |
| --- | --- | --- | --- | --- |
|  | Temporal Characteristics | Long-term, sustained, and gradual | Seconds-level fluctuations  High-frequency variations  Strong transience | Long-term, sustained |
|  | Mechanical Source | Collagen crosslinking  activation of cancer-associated fibroblasts  tumor cell-generated stretching and traction  physical constraints caused by tumor expansile growth  endothelial barrier between cells | Vascular diameter and geometry  blood flow velocity, and the cyclic contraction and relaxation of the heart | Organ-specific microenvironments and the shaping of the pre-metastatic niche |
|  | Typical Range of Values | ECM Stiffness: 4-30 kPa (or even higher) IFP : 4-100 mmHg | Veins: 0.5–4 dyn/cm² Capillaries: 30–95 dyn/cm² Arteries: 4–30 dyn/cm² Lymphatic vessels: 0.64–12 dyn/cm² | Brain: ~0.1–1.2 kPa Liver: ~0.4–0.6 kPa Bone: 10–30 GPa Lung: ~0.5–5 kPa Breast: ~2–3 kPa |
|  | Primary mechanical factors | ECM Stiffness  Solid Stress  Interstitial Fluid Pressure Cellular Traction Force | Blood Flow Shear Stress | Radial Compression Due to Microvascular Diameter Constriction  Endothelial Barrier Resistance  Organ-Specific Tissue Stiffness |
|  | Tumour Metastasis Stage | Primary detachment phase | Cyclic survival phase | Distant planting phase |

**Table 2. Quantitative induction of mechanical signals, nuclear mechanical conduction, and experimental models in cancer extravasation and colonization**

| **Table 2** Quantitative induction of mechanical signals, nuclear mechanical conduction, and experimental models in cancer extravasation and colonization | Refs | [103,192,202,246–258] | | [11,27,83,110–112,117,240,242,244,245,259–273] | | |
| --- | --- | --- | --- | --- | --- | --- |
|  | Experimental model | Microfluidic vascular models（Ex vivo）  Zebrafish models（In vivo）  Chicken chorioallantoic membrane models（In vivo） | | 3D Simulated Brain Stiffness Matrix Gel（0.5 mg/ml Collagen Ⅰ + 3 mg/ml Matrigel+ 3.3 mg/ml Hyaluronic Acid） | biomimetic liver tumor-on-a-chip model(decellularized liver matrix (DLM)+microfluidic chip) | (Adjustable aperture/stiffness+PDA surface modification) |
|  | Primary effect | 1. Transient changes in nuclear stiffness and morphology   2. Activation of nuclear membrane rupture and repair mechanisms  3 Nuclear localization of transcription factors  4 Adapt to mechanical signals within blood vessels to survive. | | 1. Persistent alterations in nuclear rigidity and morphology  2. Nuclear localisation of transcription factors  3. Mechanisms of epigenetic remodelling  4. Initiation of mechanical memory enabling organ implantation | | |
|  | Measurement Method | Ultrasound Doppler / Vector Flow Imaging（In vivo）  Phase‑contrast MRI + Wall Shear Stress Quantification（In vivo） | Multiphoton microscopy imaging, confocal microscopy imaging + software measurement（Ex vivo） | Magnetic resonance elastography（In vivo）  AFM micro-indentation（Ex vivo） | Magnetic resonance elastography（In vivo）  Ultrasound shear wave elastography （In vivo）  AFM micro-indentation（Ex vivo） | Uniaxial compression / tension（Ex vivo）  Three-point bending（Ex vivo）  Nanoindentation（Ex vivo） |
|  | Quantitative parameters | Shear stress during cycling: 0.5–30 dyn/cm² | Microvascular diameter: 4–10 μm (smaller than CTC diameter ~7–30 μm) | ~0.1–1.2 kPa | 0.4–0.6 kPa | 10–30 GPa |
|  |  |  |  | Brain | liver | bone |
|  | Primary mechanical factors | Blood flow shear stress | Localised compression of the microvasculature | Target organ tissue stiffness | | |
|  | Tumour Metastasis Stage | extravasation | | colonization | | |

**Table 3. Clinical strategies for targeting cell nuclear in treating cancer metastasis**

| Table 3 Clinical strategies for targeting cell nuclear in treating cancer metastasis | RefsClinicalTrials.gov ID | [288] | | [289] | [289] | ^[290,291]^  NCT00773474 | | | [292] | | ^[293]^  NCT01071018 | |
| --- | --- | --- | --- | --- | --- | --- | --- | --- | --- | --- | --- | --- |
|  | research phase | in vitro / in vivo | | in vitro | in vitro | in vitro / in vivo/Phase II clinical | | | in vitro / in vivo | | in vitro / in vivo/Phase I | |
|  | Mechanism of Action | Targets lamin B1 and disrupts the nuclear lamina structure, weakening the proliferation, invasion, and tumorigenicity of pancreatic cancer cells. | | Promotes the accumulation of prelamin A and upregulates the expression of mature lamin A by inhibiting HMG-CoA reductase and interfering with the farnesylation process of the precursor protein prelamin A. This significantly reduces the migration and invasion abilities of tumor cells. | Increases the expression of the LMNA gene and the levels of lamin A/C, and reduces cell migration. | Leads to the accumulation of prelamin A by inhibiting its farnesylation, and upregulates the expression of mature lamin A. Studies have shown that Lonsurf can synergize with KRAS-G12C inhibitors to enhance the anti-tumor effect. | | | Stabilizes the G-quadruplex structure in the promoter region of LINC00273 and inhibits its expression, thereby significantly reducing the migration and invasion abilities of tumor cells. | | Inhibits the phosphorylation of lamin A at Ser390 by AKT2, thereby suppressing nuclear deformation and genomic instability, restoring the integrity of the nuclear skeleton and inhibiting the migration and invasion abilities of tumor cells. | |
|  | Cancer Type | Pancreatic cancer | | Ewing's sarcoma | Ewing's sarcoma | Cervical cancer | | | Multiple cancers | | Lung cancer | |
|  | Target | lamin B1 | | lamin A/C | lamin A/C | lamin A/C | | | Link Complex | | Lamin A/C | |
|  | Drug name | Betulinic acid | | Mevinolin | 5-Azacytidine | Lonafarnib/ Lonafarnib+KRAS-G12C | | | A salt molecule of a bis Schiff base, M2 | | MK2206 | |
|  | Belongs to | Targeted modulation of sensitive structures in nuclear mechanics | | | | | | | | | | |
| Table 3 (continued) | RefsClinicalTrials.gov ID | ^[294]^  NCT02872064 | | [295–297] | [298] | | [299] | | | | [300] | |
|  | research phase | in vitro / in vivo/Phase I/IIa | | in vitro | in vitro | | in vitro | | | | in vitro / in vivo | |
|  | Mechanism of Action | Inhibits YAP-mediated tumor proliferation and metastasis by eliminating the interaction between YAP and TEAD | | Inhibits protein phosphatases PP1 and PP2A, increasing the phosphorylation of YAP/TAZ and transferring YAP/TAZ to the cytoplasm, thereby inhibiting tumor metastasis. | Inhibits YAP-mediated TEAD transcriptional activity, reducing the expression of downstream target genes, and decreasing the migration and invasion of tumor cells. | | Specifically inhibits the spontaneous palmitoylation of TEAD1-4, leading to unstable degradation of TEAD, thereby suppressing the transcriptional activity of YAP and inhibiting tumor proliferation, colony formation, and migration. | | | | Exerts anti-tumor effect by inhibiting the palmitoylation of TEAD and blocks the transcriptional activity of YAP/TEAD. | |
|  | Cancer Type | Breast cancer | | - | Mesothelioma | | Mesothelioma, breast cancer, and ovarian cancer | | | | Mesothelioma, head and neck cancer | |
|  | Target | YAP | | YAP/Taz | YAP/Taz | | YAP/Taz-TEAD | | | | YAP/Taz-TEAD | |
|  | Drug name | Verteporfin | | Okadaic acid | CA3 | | JM7 | | | | TY-0584 | |
|  | Belongs to | Targeted regulation of nuclear-related transcription factors | | | | | | | | | | |
| Table 3 (continued) | RefsClinicalTrials.gov ID | [301] | | [302] | [303,304] | [304] | | [305] | | ^[306]^  NCT04497116 | | ^[307]^  NCT04266912 |
|  | research phase | in vitro / in vivo | | n vitro / in vivo | n vitro | n vitro | | n vitro | | in vitro / in vivo/Phase I/IIa | | in vitro / in vivo/Phase I/II |
|  | Mechanism of Action | Inhibits p-YAPY357 to suppress YAP activation and nuclear localization, thereby inhibiting tumor occurrence and progression. | | Promotes the specific degradation of the TWIST1-E2A heterodimer, inhibits the stability and nuclear activity of TWIST1, and reduces tumor metastasis. | Combines the nuclear localization signal region of MRTF-A and blocks its interaction with importin α/β1, thereby inhibiting nuclear transport of MRTF-A. | | | | | May induce tumor cell death and thereby inhibit tumor metastasis by enhancing DNA damage. | | Leads to the accumulation of DNA damage by interfering with DNA damage detection, reduces the vitality of tumor cells, and thus may inhibit metastasis. |
|  | Cancer Type | Cholangiocarcinoma | | Lung cancer | Melanoma | Melanoma | | Melanoma | | Multiple cancers | | Multiple cancers |
|  | Target | YAP | | TWIST1 | MRTF-A | MRTF-A | | MRTF-A | | ART | | ART |
|  | Drug name | SHPTK2 | PND1186 | Harmine | CCG-1423 | CCG-203971 | | CCG-222740 | | Camonsertib (RP-3500) | | Berzosertib |
|  | Belongs to | Targeted regulation of nuclear-related transcription factors | | | | | | | | | | |

| Table 3 (continued) | RefsClinicalTrials.gov ID | ^[308]^  NCT04902586 | ^[309]^  NCT00008437 | ^[310]^  NCT04796220 | [311] | [312] |
| --- | --- | --- | --- | --- | --- | --- |
|  | research phase | in vitro / in vivo/Phase II | in vitro / in vivo/Phase II | in vitro / in vivo/Phase I | in vitro | in vitro |
|  | Mechanism of Action | Interferes with mitosis, induces nuclear membrane rupture, activates related signaling pathways, and inhibits cell migration and invasion. | Nuclear membrane rupture is induced by thermal effect and mechanical force, which leads to DNA damage and inhibition of metastasis. | The ultrasound and the chemically induced stress significantly upregulate the intranuclear stress signals of cancer cells, inducing apoptosis and reducing the metastatic phenotype. | It destroys the cytoskeleton of cancer cells, leading to the loss of nuclear membrane integrity and activating the cGAS-STING immune pathway, thereby inhibiting tumor metastasis. | Gold nanoparticles with nuclear localization signals can accumulate near the nuclear membrane, induce an increase in the expression of lamin A/C, enhance the mechanical strength of the nucleus, and thereby inhibit the migration and invasion abilities of cancer cells. |
|  | Cancer Type | Multiple cancers | Multiple cancers | Prostate cancer | Prostate cancer | Ovarian cancer |
|  | Target | Microtubules, nuclear membrane, cytoskeleton | Nucleus membrane, DNA repair pathway | Apoptosis-related pathways in the nucleus | Cytoskeleton, cGAS-STING、Nuclear membrane | Lamin A/C |
|  | Drug name | Tumor electric field stimulation | High-intensity focused ultrasound | Focused ultrasound and sensitizing chemicals | Low intensity pulsed ultrasound | Gold nanoparticles targeting the nuclear membrane |
|  | Belongs to | Physiotherapy | | | | |
